# Comparative transcriptome analysis of the interaction between *Actinidia chinensis* var. *chinensis* and *Pseudomonas syringae* pv. *actinidiae* in absence and presence of acibenzolar-S-methyl

**DOI:** 10.1186/s12864-018-4967-4

**Published:** 2018-08-06

**Authors:** Vania Michelotti, Antonella Lamontanara, Giampaolo Buriani, Luigi Orrù, Antonio Cellini, Irene Donati, Joel L. Vanneste, Luigi Cattivelli, Gianni Tacconi, Francesco Spinelli

**Affiliations:** 1 Council for agriculture research and economics (CREA), Research Centre for Genomics and Bioinformatics, via S. Protaso, 302, CAP, 29017 Fiorenzuola d’Arda, Piacenza Italy; 2 Department of Agricultural Sciences Alma Mater Studiorum, University of Bologna, viale Fanin 46, 40127 Bologna, Italy; 3 The New Zealand Institute for Plant & Food Research Ltd, Ruakura Research Centre, Bisley Road, Ruakura, Private Bag 3123, Hamilton, 3240 New Zealand

**Keywords:** *Actinidia chinensis*, *Pseudomonas syringae* pv. *actinidiae*, RNAseq, Acibenzolar-S-methyl, Hormonal balance (HB), Transcription factors (TFs)

## Abstract

**Background:**

Since 2007, bacterial canker caused by *Pseudomonas syringae* pv. *actinidiae* (Psa) has become a pandemic disease leading to important economic losses in every country where kiwifruit is widely cultivated. Options for controlling this disease are very limited and rely primarily on the use of bactericidal compounds, such as copper, and resistance inducers. Among the latter, the most widely studied is acibenzolar-S-methyl. To elucidate the early molecular reaction of kiwifruit plants (*Actinidia chinensis* var. *chinensis*) to Psa infection and acibenzolar-S-methyl treatment, a RNA seq analysis was performed at different phases of the infection process, from the epiphytic phase to the endophytic invasion on acibenzolar-S-methyl treated and on non-treated plants. The infection process was monitored in vivo by confocal laser scanning microscopy.

**Results:**

De novo assembly of kiwifruit transcriptome revealed a total of 39,607 transcripts, of which 3360 were differentially expressed during the infection process, primarily 3 h post inoculation. The study revealed the coordinated changes of important gene functional categories such as signaling, hormonal balance and transcriptional regulation. Among the transcription factor families, AP2/ERF, MYB, Myc, bHLH, GATA, NAC, WRKY and GRAS were found differentially expressed in response to Psa infection and acibenzolar-S-methyl treatment. Finally, in plants treated with acibenzolar-S-methyl, a number of gene functions related to plant resistance, such as PR proteins, were modulated, suggesting the set-up of a more effective defense response against the pathogen. Weighted-gene coexpression network analysis confirmed these results.

**Conclusions:**

Our work provides an in-depth description of the plant molecular reactions to Psa, it highlights the metabolic pathway related to acibenzolar-S-methyl-induced resistance and it contributes to the development of effective control strategies in open field.

**Electronic supplementary material:**

The online version of this article (10.1186/s12864-018-4967-4) contains supplementary material, which is available to authorized users.

## Background

Kiwifruit is an economically important crop in several countries such as New Zealand, Italy, Chile, Iran and China, with Italy and New Zealand being the largest exporters of kiwifruit [1]. However, since 2007, kiwifruit industries suffered from a global outbreak of bacterial canker caused by the Gram-negative bacterium *Pseudomonas syringae* pv*. actinidiae* (Psa) [2, 3]. Psa enters plants through stomata or wounds and successively colonizes the vascular system, and it can spread systemically to young twigs a few minutes after penetration [4]. Molecular studies on Psa populations identified five biovars named biovar 1, 2, 3, 5 and 6 [3, 5–7]. Biovar 4 is now classified as a different pathovar called *actinidifoliorum* [8]. Biovar 3 is the biovar that caused the global outbreak [7]. Biovar 3 strains are characterized by the presence of pathogenesis-related sequences (integrative conjugative elements, ICEs), horizontally acquired from other *P. syringae* pathovars [9], but do not produce any known toxin. The control of this disease is primarily based on the use of bactericidal foliar copper application and on the induction of natural plant resistance [10, 11]. acibenzolar-S-methyl is among the most effective resistance inducers and it is able to activate Systemic Acquired Resistance (SAR) in kiwifruit plants [12]. Plants possess two levels of defense mechanisms against pests and pathogens: the general Pathogen associated molecular Pattern-Triggered Immunity (PTI) and the more specific Effector-Triggered Immunity (ETI), which is initiated after recognition of specific pathogen effector proteins [13]. Plants have evolved surveillance mechanisms that are activated upon recognition of “non-self” (or “damaged-self”) molecular patterns or signals by cell surface located receptors called Pattern Recognition Receptors (PRRs) [14]. Acute PRR signaling results in the accumulation of Reactive Oxygen Species (ROS), activation of ion channels and of defense-related Mitogen-Activated Protein Kinases (MAPKs), and transcriptional reprogramming [15]. Transcriptional reprogramming, driven by specific transcription factors (TFs), is a main feature of plant response to biotic stresses [16]. Specific members of the major TF families (AP2/ERF, bHLH, bZIP, MYB, NAC and WRKY) are particularly committed to regulating plant responses to pathogens. Furthermore, a cross-talk occurs among a core of TFs and genes related to phytohormone biosynthesis and signaling. Phytohormones, such as jasmonic acid (JA) ethylene (ET) and salicylic acid (SA), are signaling molecules that regulate the main part of plant response to pathogens [17] and TFs are involved in the convergence of different plant hormonal signaling pathways [18]. Often JA and ET work synergistically to trigger resistance against necrotrophic pathogens, whereas SA acts on the resistance against biotrophic ones. SA and JA/ET defense pathways are reciprocally antagonistic, therefore an elevated resistance against biotrophs is often correlated with susceptibility to necrotrophs, and vice versa [19, 20]. Many pathogens have evolved sophisticated strategies to manipulate the plant hormonal balance. The best-characterized mechanism involves the bacterial phytotoxin coronatine (COR), an analog of methyl-JA, which causes both the repression of the SA defense pathway and stomata opening, conditions which facilitate bacterial colonization of host tissues [21].

Little is known on the molecular interactions between *A. chinensis* var. *chinensis* and Psa. Up to very recently, how infection by Psa or treatment by elicitors of host resistance modifies gene expression in *Actinidia sp.* was still mostly unexplored. It has been demonstrated that elicitors of the SA pathway, but not of the ET/JA pathway, limit disease severity in both *A. chinensis* var. *chinensis* and *A. chinensis* var. *deliciosa* [12], and that following elicitation the genes PR1, PR8, ICS and PAL were over expressed in *A. chinensis* var. *deliciosa* and to lesser extent in *A. chinensis* var. *chinensis* [12]. Elicitation by chitosan also lead to the overexpression of the genes PR1 and PR5 [22]. The genes β 1–3 glucosidase, WRKY40, PR6 and AP2ERF2 were found overexpressed after infection by Psa [23] while analysis of the transcriptome of *A. deliciosa* found that several WRKY genes as well as several resistance genes (RPS2 and RPM1) were upregulated after inoculation with Psa [24]. An analysis of the WRKY genes of *Actinidia* species lead to the discovery of 97 genes which could be grouped in three categories [25]. After inoculation with Psa several of those WRKY genes were overexpressed (e.g. WRKY38, and WRKY 95) while some were repressed (e.g. WRKY96) [25]. It is now known that Psa infection results in massive changes of the transcriptome and that different species of Actinidia react differently to the infection [24]. Comparison of the transcriptome of *A. chinensis* var. *chinensis*, *A. arguta* and *A. eriantha* lead to the suggestion that resistance to Psa was related to the expression of a number of long non-coding RNAs that act in concert with coding genes [24]. To date, no comparison has been done of the whole transcriptome after inoculation of elicited versus non-elicited plants. This study aimed to compare gene expression in *A. chinensis* var. *chinensis* plants after inoculation with Psa, on plants which were elicited or not.

Comparative transcriptome profiling of *A. chinensis* var. *chinensis* in response to Psa was carried out to describe the molecular response of leaf cells to Psa and the molecular mechanisms underlying the increased resistance observed after the exogenous application of acibenzolar-S-methyl. The transcriptome analysis was tailored to the different steps of the infection process, from the epiphytic phase until the invasion of the host tissues. The progression of the infection was monitored in vivo by fluorescent stereomicroscopy and confocal laser scanning microscopy. Along with de novo assembly and characterization of the transcriptome, analysis of global patterns of gene expression and functional categorization was performed. Knowledge of the mechanisms involved in the acquired resistance of kiwifruit plants after the acibenzolar-S-methyl application could be useful to assist in identifying molecular markers in breeding for resistance.

## Methods

### Plant material

In vitro rooted plantlets of *A. chinensis* var. *chinensis* from micropropagation, 5–8 cm shoot length, were used for the experiment. The plants were grown in 1000 mL plastic jars containing 200 mL of half-strength Murashige and Skoog basal medium basal medium without sucrose [26]. The plants were left for 15 days in these jars before starting the experiments. For the whole duration of the experiment the plants were kept in a growing chamber at 22 ± 1 °C, with a 16:8 h light:dark period.

### Acibenzolar-S-methyl treatment and Psa infection

The experiment was carried out with plants treated with acibenzolar-S-methyl or with water 15 days before inoculation with Psa, or mock inoculation using buffer. Acibenzolar-S-methyl-treated plants were immersed for 5 s in sterile solution 1.7 mM acibenzolar-S-methyl (Bion®50WG, Syngenta, Basel, Switzerland), whereas the untreated plants were submerged in water, 15 days before Psa inoculation [12]. The Psa strain CFBP7286-GFPuv was used for inoculation [27]; the inoculum was prepared from 48 h grown colonies resuspended in 10 mM MgSO_4_ to a final concentration of 10^8^ cfu/mL. Inoculation was performed by immersing the plants in the Psa suspension for 1 min. The mock-inoculated plants were immersed in sterile 10 mM MgSO_4_. The Psa-inoculated plants were indicated as “Inoculated” (I), the mock inoculated plants were referred as “Healthy Control” (HC), the plants treated with acibenzolar-S-methyl only were indicated as “ASM” (ASM) and the plants treated with acibenzolar-S-methyl and then inoculated with Psa were indicated as “ASM-Inoculated” (ASM.I). Samples for RNA-seq analysis were taken 3, 24 and 48 h post-inoculation (hpi) and frozen by dipping in liquid nitrogen. The experiment was carried out with five biological replicates of five plants each: two replicates were used for transcriptome analysis according to recommended RNA-seq standards (Encode project https://genome.ucsc.edu/ENCODE/protocols/dataStandards/ENCODE_RNAseq_Standards_V1.0.pdf), one was kept till the development of the symptoms to assure the effectiveness of the inoculation, one was employed to assess Psa colonization and one was used for the confocal laser scanning microscope analysis.

### Assessment of Psa colonization

Disease incidence and severity were assessed according to Cellini et al., 2014 [12]. Epiphytic and endophytic Psa populations were monitored in the first 48 h post inoculation. For this purpose, plants were washed in 10 mL of sterile 10 mM MgSO_4_ under gentle agitation for 15 min, then the washing solution was serially diluted and each dilution was plated in triplicate to evaluate Psa epiphytic population as cfu/mL. To assess the endophytic Psa population, the plants, washed as above, were externally sterilized by dipping each of them for 1 min in 70% ethanol, then 1 min in 1% sodium hypochlorite followed by 2 min in sterile water. After this treatment, the plants were homogenized in 10 mL of sterile 10 mM MgSO_4_. The homogenate was 10 folds serially diluted and each dilution was plated in triplicate as previously described. Bacterial colonies were enumerated at 0, 3, 6, 12, 24 and 48 h after inoculation and Psa population was calculated as cfu g^− 1^ of plant fresh weight.

### Real time monitoring of the colonization of the host tissues

In order to match the transcriptome analysis to the different steps of the infection process, from the epiphytic colonization until the invasion of the host tissues, the infection was monitored in vivo by fluorescent stereomicroscopy and confocal laser scanning microscopy. At 3, 6, 12, 24 and 48 h post inoculation, leaves were initially observed with a Nikon SMZ25 fluorescence microscope (Nikon Instruments Corporation, Tokyo, JAPAN), with an optical system providing a zoom ratio of 25:1 (zoom range 0.63 x - 15.75 x) and epifluorescence filter cube selection (excitation wavelength of GFP-B: 460–500 nm, emission wavelength: 510–560 nm). Once Psa colonization was located on the leaf lamina, further observations were performed using a NIKON C1-S confocal laser scanning microscope equipped with an Argon laser. Optical sections of leaf lamina were acquired at 40, 60 and 100 x with Nikon PlanApo objectives and the BHS (GHS) filter set. Images were acquired and analysed by the NIS-Elements C Microscope Imaging Software.

### RNA extraction and libraries preparation

Total RNA was extracted from 100 mg of ground tissue using Plant/Fungi Total RNA Purification kit (NorgenBiotek Corp., Canada) following manufacturer’s instructions. RNA samples were treated with RNase-free DNase I (Ambion, TX, USA) to remove contaminating DNA. Purity and concentration of the samples were estimated with a spectrophotometer. The integrity of the RNA (RIN > 8) was evaluated on an RNA 6000 Nano LabChiprun on Agilent 2100 Bioanalyzer (Agilent Technologies, Germany). Only samples with a RIN over 8 were used for the experiment. Two micrograms of total RNA were subjected to library preparation using the TruSeq RNA sample preparation v2 kit (Illumina, San Diego, CA, USA) following the manufacturer’s instructions. Libraries were amplified by 15 cycles of PCR and then size selected for an average size of 300 bp using a 2% low range ultra-agarose gel (BIO-RAD). Library quality and size were assayed on the Agilent 2100 Bioanalyzer.

### Sequencing, raw reads processing and data analysis

Libraries were paired end sequenced for 75 bp using an Illumina Genome Analyser (GAIIx) generating about 730 millions of raw reads. FastQ file generation was performed by CASAVA v1.8. Raw Illumina reads were processed using the CLC Genomics Workbench software (CLCbio, Aarhus, Denmark) to remove low-quality reads, adapters and duplicated sequences. Transcriptome de novo assembly was performed using Trinity software [28] using the following parameters: fixed default k-mer size 25, minimum contig length 200 bp and minimum k-mer coverage 3. Redundant sequences present in the assembly were removed using CD-HIT software [29] with a similarity threshold of 90%. Moreover, the contigs were blasted against *Pseudomonas syringae* genomes (http://www.pseudomonas-syringae.org) and the contigs showing a significant match were removed.

Contigs annotation was produced with Blast2GO [30] searching for matches against the NCBI non-redundant protein database, RefSeq protein, Swiss-Prot/Uniprot database, NCBI non-redundant nucleotide database, InterPro database and the Kiwifruit Genome database (http://bioinfo.bti.cornell.edu/cgi-bin/kiwi/download.cgi) with a E value threshold of 1e^− 5^ and by using the functionality of InterProScan v5.0 that allowed retrieval of domain/motif information in the InterPro as well as in other domain databases. Furthermore, local BLASTX alignments were run against the Clusters of Orthologous Groups (COGs) database and the Kiwifruit Genome database [31]. Reads were mapped to the assembled contigs and counted using the CLC software. Differentially expressed genes (DEG) were determined using the R package DESeq [32]. Gene Ontology enrichment analysis was performed using the GOseq package [33]. For a complete functional annotation of the transcriptome, a local BLASTx was performed to query the kiwifruit plant protein databases obtained from the Kiwifruit Genome database.

### MapMan, KEGG tools and WGCNA

MapMan figures were obtained with the Mercator tool, using default parameters (http://mapman.gabipd.org/web/guest/mercator), to assign MapMan bins to Kiwifruits transcripts [34]. Log2 fold changes as obtained from DESeq output were used as MapMan input to represent expression changes. The KASS software was used to generate the KEGG (Kyoto Encyclopedia of Genes and Genomes) pathway picture. The KEGG database integrates genomic information with higher order functional information by collecting manually drawn pathway maps representing current knowledge on cellular processes and standardized gene annotations [35].

Gene expression was also assessed with weighted-gene co-expression network analysis (WGCNA) [36]. To analyse networks co-expression, transcripts were filtered for normalized count > 10 and the genes that have a high percentage of missing counts (> 90%) were removed, leading to a total number of 27.384 transcripts. The pickSoftThreshold was used to select the lower power for which the scale-free topology fit index curve flattened out upon reaching a high value. The weighted adjacency matrix of a signed network was obtained at power 9 (R function adjacency). Hierarchical clustering was conducted using the R package flashClust and the cuttreeDynamic function (dendro = geneTree, distM = dissTOM, method = “hybrid”, deepSplit = 2, pamRespectsDendro = F, minClusterSize = 30) were employed to identify modules. These functions have been shown to be the best approach for biologically meaningful results [37].

### Validation of DEGs by qRT-PCR

The transcription of twenty DEGs was determined using quantitative real time PCR (qRT-PCR). Primers were designed using Primer3 plus Software (http://www.bioinformatics.nl/cgi-bin/primer3plus/primer3plus.cgi) and their specificity was checked by blasting their sequences against the NCBI database. The genes employed in the validation experiment and the primer information are reported in Additional file 1: Table S1.

Samples were collected from three biological replicates in an experiment conducted in the same conditions as the one used for the RNA-seq analysis. Total RNA was treated with RNase-free DNase I (Ambion) to remove the contaminating DNA and the first cDNA strand was synthesized from 1.0 μg of total RNA by reverse transcription using Superscript II (Invitrogen, Life Technologies GmbH, Darmstadt, Germany).

Real-time quantification was performed using the ABI 7300 Real Time System (Applied Biosystems). KAPA Sybr Fast qPCR kit (Resnova) master mix was used for 40 cycles with the following profile: 95 °C for 15 s, 60 °C for 20 s, 72 °C for 40 s. Melting curve analysis was performed to verify single product amplification with temperature ranging from 55 to 95 °C by increasing of 1 °C every step. All reactions were run in triplicate for each biological replicate and β-Actin (ACT1, accession number EF063572) was used as the reference gene. Primers for β-Actin (Additional file 1: Table S1) were designed by Primer 3 plus (http://www.bioinformatics.nl/cgi-bin/primer3plus/primer3plus.cgi) based on complete mRNA sequence of *Actinidia deliciosa* ACT1 (ACT1), accession number EF063572. Transcript abundances are given as the mean ± SE of replicates. Relative transcription levels were calculated using the 2-△△CT method [38].

## Results and discussion

### Host tissues colonization

**Fig. 1:**
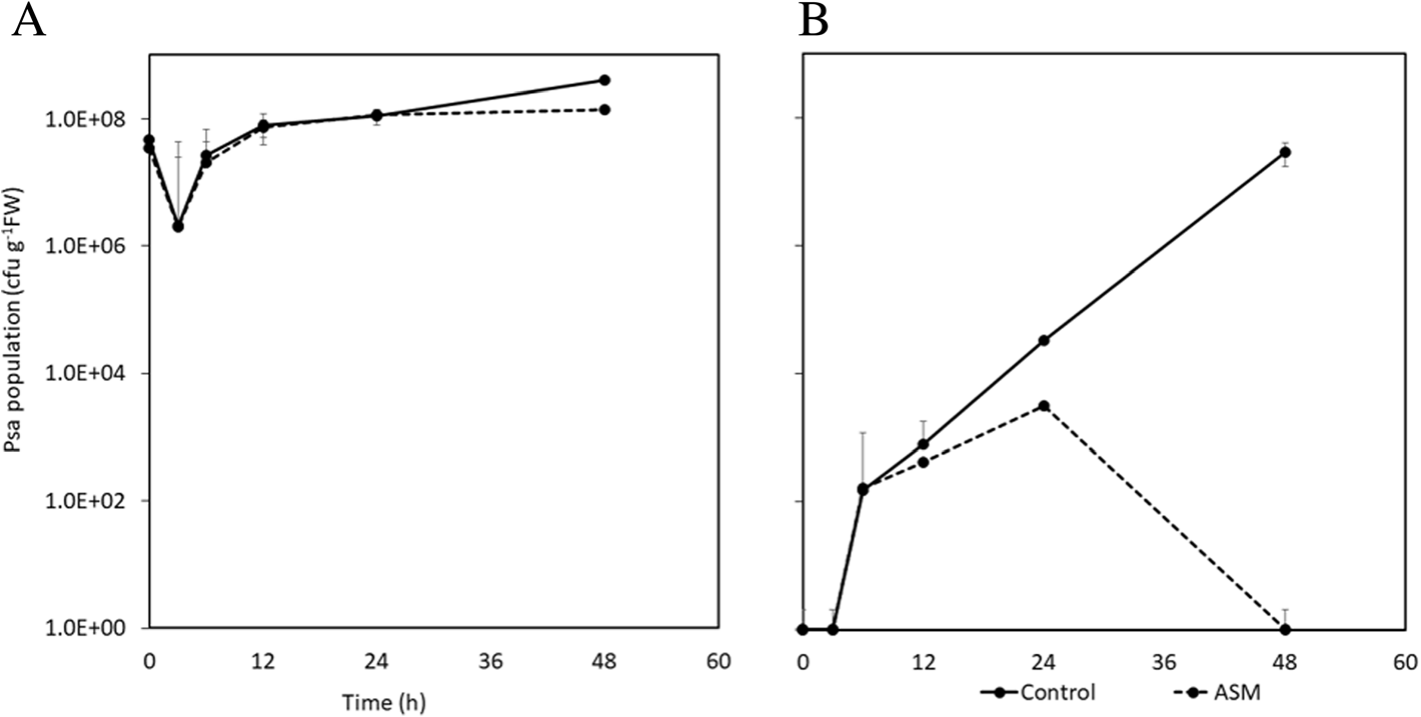
Epiphytic (**a**) and endophytic (**b**) colonization of *Actinidia chinensis* var. *chinensis* plants by *Pseudomonas syringae* pv. *actinidiae* (Psa) strain CFBP7286-GFPuv at 3, 6, 12, 24 and 48 h after inoculation. Plants were either treated with water (Control) or acibenzolar-S-methyl application (ASM) 15 days before inoculation

Psa epiphytic population reached their maximum concentration about 6 h post-inoculation (hpi), a result determined by the high inoculum used in this experiment and by Psa epiphytic fitness (Fig. 1a). The acibenzolar-S-methyl treatment did not influence the epiphytic growth on host leaves. From 6 hpi onwards, Psa was also found inside the host tissues (Fig. 1b), although in acibenzolar-S-methyl treated plants, Psa growth was reduced in comparison with non-treated plants. Furthermore, from 24 hpi onwards, Psa endophytic populations decreased in acibenzolar-S-methyl treated plants reaching less than 10 cfu g^− 1^ fresh tissues at 48 hpi (Fig. 1b).

**Fig. 2:**
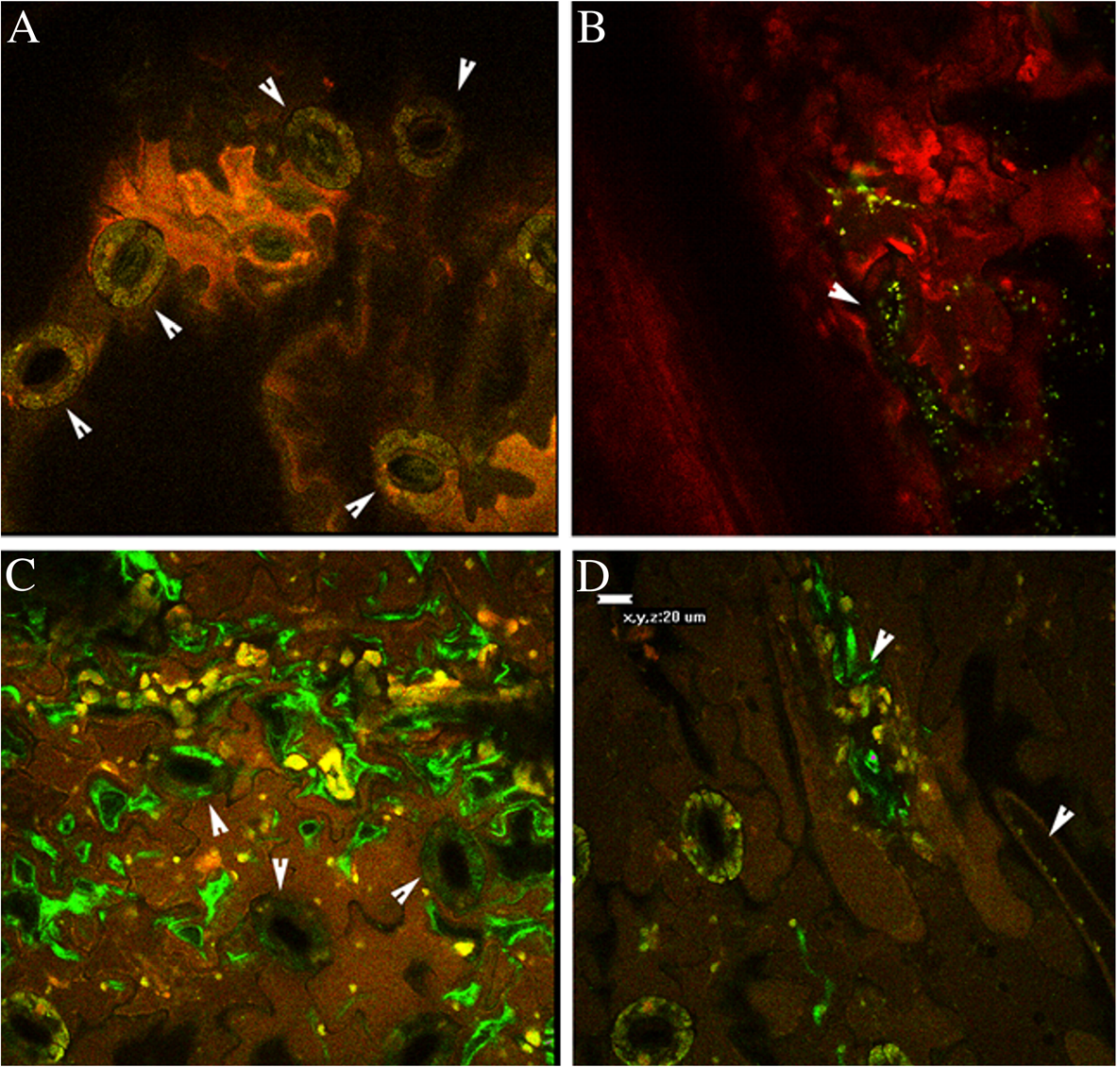
Confocal laser scanning micrographs of *Actinidia chinensis* var. *chinensis* leaves inoculated with *Pseudomonas syringae* pv. *actinidiae* Psa strain CFBP7286-GFPuv. Panel **a**: before infection, white arrows indicate stomata; Panel **b**: 6 h after infection. It is possible to observe Psa cells colonizing the leaf surface and the stomata entrance (arrows); Panel **c**: 24 h after infection, vertical sectioning of leaf lamina (Z axis: - 20 μm) showing Psa invading the spongy mesophyll below stomata (arrows); Panel **d**: 24 h after infection. Psa colonizing the leaf veins (arrows)

Observations under a confocal laser scanning microscopyconfirmed the dynamic of Psa colonization. At 6 hpi, Psa was localized around the stomata and on the edges of the stomata among epidermic cells (Fig. 2b). At 24 hpi, Psa was clearly observed inside the stomatal chamber and in the spongy mesophyll, indicating that the bacterium rapidly entered the host tissues via the stomata and extensively colonized the leaves (Fig. 2c). The assessment of the endophytic population indicated that Psa had already entered the leaves at 6 hpi; however, Psa was visible by confocal laser scanning micrographs only between 12 and 24 hpi, suggesting that a certain population threshold was needed to make colonization visible. At 24 hpi, colonization of secondary and tertiary leaf veins was observed (Fig. 2d), suggesting the beginning of systemic invasion of the kiwifruit vine.

### RNA sequencing and transcriptome assembly

After quality filtering, between 11 and 46 million reads were obtained for each RNA sample (Additional file 2: Table S2 A). Pearson correlations between biological replicates were always above 0.95 and samples undergoing the same treatment clustered together. High-quality reads were used to produce the reference transcriptome of *A. chinensis* var. *chinens*is by de novo assembly. The overall transcriptome consisted of 63,943 contigs. Then, redundant contigs, as well as those belonging to *P. syringae*, were removed leading to 39,584 contigs, for a total assembly size of 39.95 Mbp. The sequence length ranged from 201 bp to 11,870 bp with an average size of 933 bp and a N50 of 1472 bp.

### Annotation and classification of *A. chinensis* var. *chinensis* transcriptome

The main features of the annotation of the *A. chinensis* var. *chinensis* reference transcriptome are summarized in Additional file 3: Figure S1 A, while the complete information is presented in Additional file 2: Table S2 B.

The E-value distribution of the top hits for each contig in the NR protein database showed that 18,369 contigs had strong homology with an E value < 1.0 e^− 5^ (Additional file 3: Figure S1 B). The sequence similarity distribution of the contigs against the NR protein database shows that 24,867 contigs (85.93%) have a similarity ranging from 100 to 70% (Additional file 3: Figure S1 C). The BLASTx performed to query the *Actinidia* protein databases, obtained from the Kiwifruit Genome database, led to a match with 30,634 annotated contigs (77.39%).

The contigs of the reference transcriptome were classified in 13,619 Clusters of Orthologous Groups (COGs) functional annotations and grouped into 24 function categories (Additional file 3: Figure S1 D). Moreover, 21,636 contigs of the *A. chinensis* var. *chinensis* transcriptome were categorized into 101 functional groups of Groups of Orthologous (GO) slim plant (Additional file 3: Figure S1 E). As final result, a total of 34,039 contigs were annotated by integrating the BLAST results from all the database queried (Additional file 3: Figure S1 F and H, Additional file 2: Table S2 C and D).

This work identified a total of 4533 DEGs (Additional file 4: Table S3). The interaction between kiwifruit plants and Psa revealed 3360 DEGs, most of them were identified at 3 hpi (2747 DEGs). Few DEGs were highlighted at 24 hpi and at 48 hpi (272 and 341 DEGs, respectively; Table 1). The comparison between HC and Psa-inoculated plants at 3 hpi showed that 1596 DEGs were up-regulated at least two-fold, with about 37% of them being up-regulated more than five-fold and 214 more than ten-fold. About 42% of DEGs were down-regulated. At 24 hpi and 48 hpi the response was dominated by a down-regulation of gene expression with 57 and 76% of DEGs repressed, respectively. The small number of DEGs at 24 hpi and 48 hpi, and the prevalence of down-regulation may reflect the ability of Psa bacterial cells to suppress the plant defense pathways.

**Table 1:** Identification of Differentially Expressed Genes (DEGs)

	Comparison	Total DEGs	Up regulated	Down regulated
DEGs associated with the infection process	HC_vs_I3	2747	1596	1151
	HC_vs_I24	272	116	156
	HC_vs_I48	341	81	260
DEGs associated with acibenzolar-S-methyl treatment	HC_vs_ASM	819	474	345
DEGs associated with infection in acibenzolar-S-methyl treated plants	I3_vs_ASM.I3	510	323	187
	I24_vs_ASM.I24	1374	552	822
	I48_vs_ASM.I48	1252	747	505

HC was healthy control plants; I3, I24, I48 were inoculated plants at 3, 24 and 48 h post-inoculation; ASM represented acibenzolar-S-methyltreated healthy plants; ASM.I3, ASM.I24, ASM.I48 were acibenzolar-S-methyltreated plant inoculated at 3, 24 and 48 h post-inoculation (hpi)

ASM plants reacted differently to Psa infection than non-treated ones. At 3 hpi in ASM plants, a substantially lower number of DEGs (510) were modulated in comparison to untreated plants. In contrast, at 24 hpi in ASM plants, 1374 DEGs were identified and at 48 hpi, the number of DEGs was 1252 (Table 1). Noteworthy, the treatment with acibenzolar-S-methyl sustained a much stronger molecular response to Psa infection at 24 hpi and 48 hpi, in agreement with the observed decreasing of Psa population inside the leaves (Fig. 1).

**Fig. 3:**
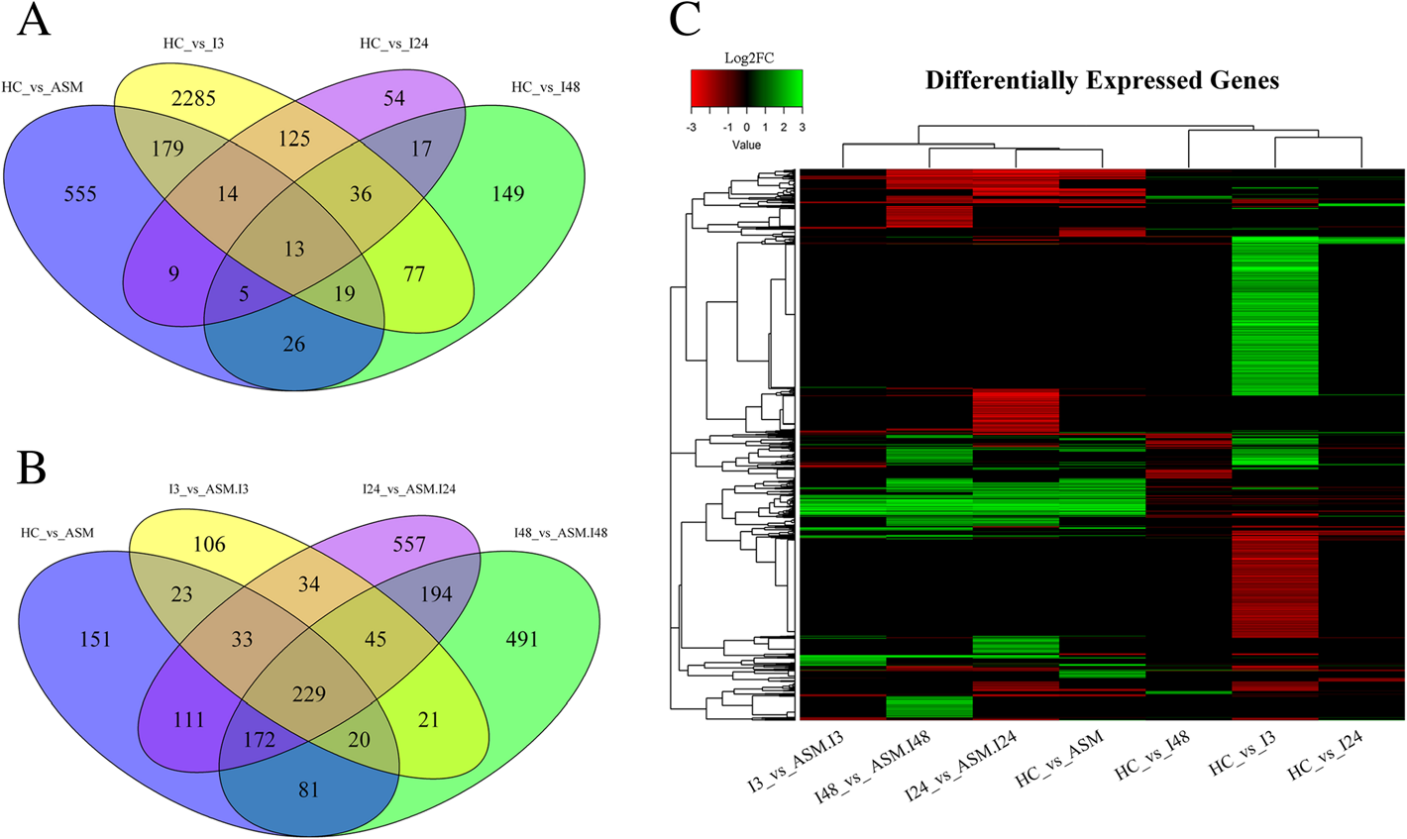
Panel **a**: Venn diagrams showing the overlapping of the DEGs modulated in response to *Pseudomonas syringae* pv. *actinidiae* (Psa) infection at 3, 24 and 48 hpi in plants not treated with acibenzolar-S-methyl. Panel **b**: Venn diagrams showing the overlapping of the DEGs modulated in response to Psa infection at 3, 24 and 48 hpi in plants untreated and treated with acibenzolar-S-methyl. Panel **c**: Hierarchical clustering analysis made with Pearson correlation of Log2FC of all DEGs detected in the transcriptome analysis (4533; Additional file 4: Table S3). Each row represents a transcript; each column represents a comparison. A dendrogram of the correlation among transcripts is shown on the left of the heatmap. A clear separation between untreated and acibenzolar-S-methyl treated plants is evident from the upper cluster of the heatmap

In untreated plants, out of 2747 DEGs detected at 3 hpi, only 188 were shared with 24 hpi, and this number decreased to 145 at 48 hpi. Acibenzolar-S-methyl treated and untreated plants shared 225 DEGs at 3 hpi, 41 at 24 hpi and 53 at 48 hpi (Fig. 3a).

In inoculated ASM plants, out of 510 DEGs modulated at 3 hpi, 341 were shared with 24 hpi and this number became 315 at 48 hpi (Fig. 3b). In ASM plants, regardless of inoculation status, a core group of 229 DEGs were detected at all-time points after inoculation. Finally, 151 DEGs were identified exclusively in non-inoculated ASM (Fig. 3b).

The hierarchical clustering analysis based on transcript expression levels had grouped the DEGs in two main clusters related to the acibenzolar-S-methyl treatment. This result indicated that acibenzolar-S-methyl strongly affected the plant response to Psa infection, suggesting that this compound activated defensive responses which were not induced in untreated plants (Fig. 3c). For the validation of our transcriptomic data twenty DEGs were selected for qRT-PCR analysis (Additional file 5: Figure S2). Our qRT-PCR data strongly correlated with the RNA-seq expression data.

### Biochemical pathways analysis

The kiwifruit transcripts modulated in response to Psa infection were analyzed by the KEGG orthology database. Among the 4533 DEGs identified in the transcriptomic analysis, 1331 were assigned to KEGG orthologs. Among them, KEGG analyses identified several pathways, such as plant hormone signal transduction, cysteine-methionine metabolism and plant-pathogen interaction. These pathways were differentially modulated according to the progression of Psa in the tissues and to presence/absence of acibenzolar-S-methyl treatment. In addition, the visualization of the DEGs with MapMan analysis highlighted the involvement of the hormonal signaling and the modulation of the transcription factors during the progression of Psa invasion and presence/absence of acibenzolar-S-methyl treatment (Additional file 6: Figure S3).

### Pathogen recognition related genes

**Fig. 4:**
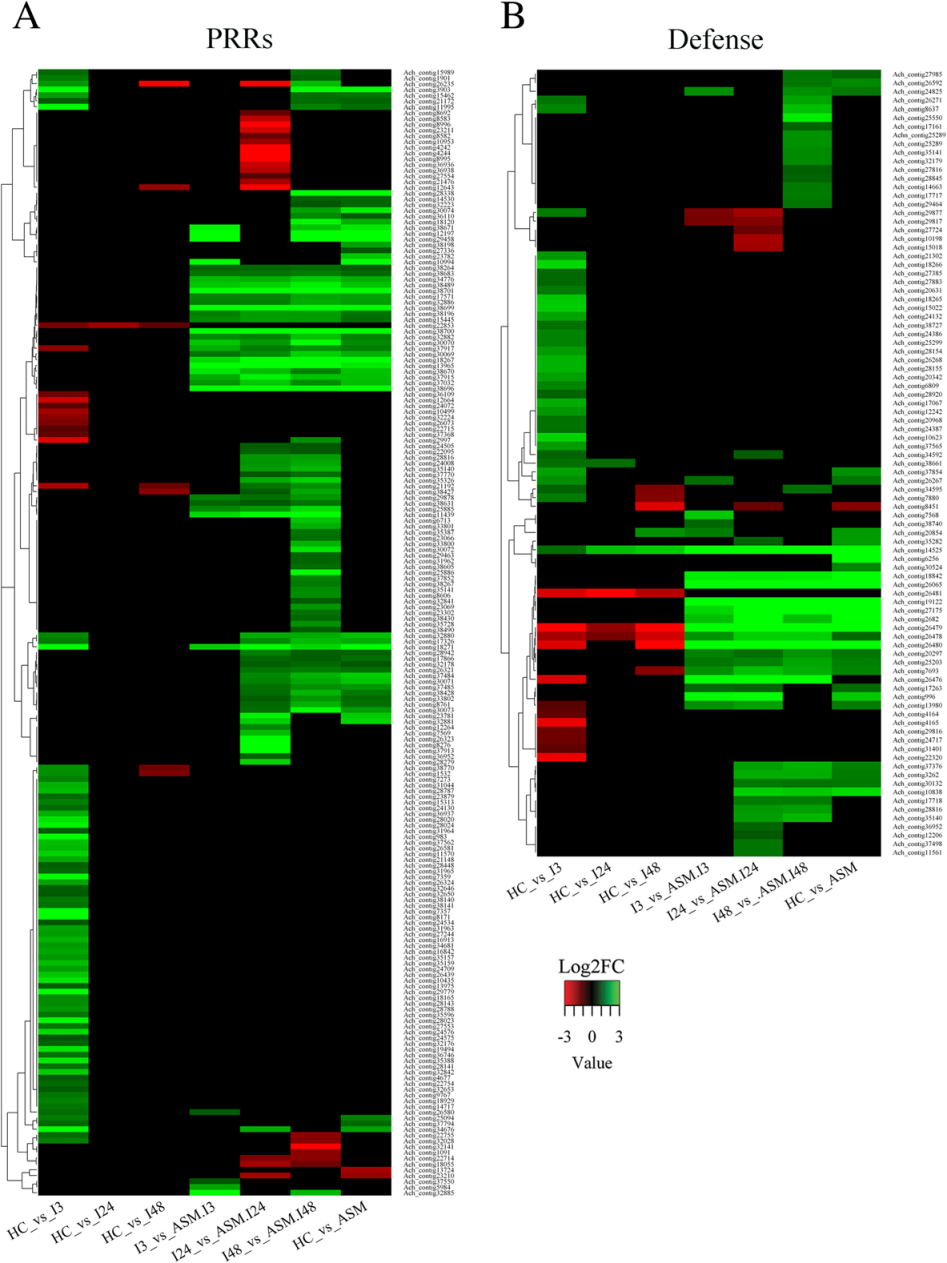
Panel **a**: Heatmap of Differentially Expressed Genes (DEGs) encoding putative Pattern Recognition Receptors (PRRs). Panel **b**: Heatmap of DEGs involved in the defense response against Psa. Up-regulated transcripts (Log2FC ≥ 1) are in green, down-regulated transcripts (Log2FC ≤ − 1) are in red. Each row represents a transcript, each column a comparison. For the description of the gene names represented in the heatmaps refers to Additional file 4: Table S3 and Additional file 8: Table S5

Perception of microbe-associated molecular patterns or pathogen-associated molecular patterns (PAMPs/MAMPs) via activation of cell-surface-resident pattern recognition receptors (PRRs) initiates the PAMP-triggered immunity (PTI) [13]. In kiwifruit plants inoculated with Psa, 198 transcripts encoding putative Pathogen Recognition-Related (PRR) proteins were found to be differentially expressed (Fig. 4a, Additional file 7: Table S4). These PRRs are Receptor Like Kinases (RLK) or Leucine-rich repeat receptor-like protein kinases (LRR-RLKs).

The most significant up-regulation of RLK and LRR-RLK transcripts was observed in inoculated, ASM plants with an increase in the number of up-regulated transcripts from 3 to 48 hpi. On the other hand, a different set of PRR transcripts were found up-regulated almost exclusively at 3 hpi in infected untreated kiwifruit plants (Fig. 4a).

Transcripts homologous to FLAGELLING SENSING 2 (Ach_contig26439; Ach_contig38670, Ach_contig38671), the receptor kinase for bacterial flagellin (flg22), were up-regulated. Ach_contig26439 was up-regulated in untreated plants only at 3 hpi, while Ach_contig38670 and Ach_contig38671 were up-regulated in ASM plants regardless of infection. In other pathosystems, the activation of flg22 leads to the accumulation of ET and SA [39–41]. Other transcripts associated with bacterial recognition were up-regulated after infection by Psa. The bacterial elongation factor Tu (EF-Tu) (Ach_contig7359), was up-regulated at 3 hpi in untreated samples only. Instead, transcripts belonging to the serine/threonine receptor kinase family, containing the LysM domain [42], were up-regulated in both acibenzolar-S-methyl-treated and untreated plants at 3 hpi (Fig. 4a). LysM domains bind Peptidoglycans (PGNs) of both Gram-positive and Gram-negative bacteria and are known to mediate resistance against bacterial pathogens [43].

Oligogalacturonidases (OGs) fragments, released from plant cell walls, act as potent defense response elicitors [44]. The WALL-ASSOCIATED KINASES (WAKs) work as pathogen-related signals molecules, which are able to detect the presence of OGs [45, 46].

In *Arabidopsis*, the increase of expression of some WAK genes e.g. WAK2, [47] or WAKL10 and WAKL22 [48] was found related to pathogen infection. Moreover, WAK1 has been linked with both to *P. syringae* infection and exogenous application of SA [49]. In rice, OsWAK25 transcript accumulates after acibenzolar-S-methyl-treatment [50], and in tomato and wheat, WAK genes have been found to be related with pathogen infection [51, 52].

In the kiwifruit-Psa interaction, 10 DEGs belonging to the WAK family (Ach_contig12264; Ach_contig3903; Ach_contig11995; Ach_contig25885; Ach_contig25886; Ach_contig35387; Ach_contig37852; Ach_contig35388; Ach_contig15313) were up-regulated in untreated infected plants at 3 hpi and as well as in ASM plants regardless of sampling time (Fig. 4a and Additional file 7: Table S4).

Members of the RLK1 family are good indicators for cell wall integrity. Among them, Theseus1 (THE1) and Feronia (FER) were differentially expressed in kiwifruit-Psa interaction [53, 54]. Transcripts annotated as receptor-like kinase FERONIA were up-regulated only in ASM plants regardless of infection (Fig. 4a). Among them, Ach_contig13965 was used to confirm these data by qRT-PCR (Additional file 5: Figure S2). In response to environmental stress and SA, FER receptor kinase may negatively regulate the biosynthesis of S-adenosyl methionine (SAM) which, in turn, down-regulates the SAM-dependent pathways, such as the one leading to ET biosynthesis [55].

BRASSINOSTEROID-INSENSITIVE ASSOCIATED KINASE 1 (BAK1) is required for responses triggered by the orphan PAMPs such as Peptidoglycans (PGNs) and lipopolysaccharide (LPS) [56] Moreover, BAK1 is essential for PRRs for the responses against different bacterial and fungal pathogens [57]. Three transcripts homologs to BAK1 were found differentially expressed in kiwifruit after Psa interaction. Ach_contig24575 and Ach_contig24576 were up-regulated in infected plants at 3 hpi only, while Ach_contig29878 was up-regulated in acibenzolar-S-methyl-treated and inoculated samples, at all-time points. The expression of the BAK1-Ach_contig29878 reflects the ability of ASM plants to activate the downstream immune response (Fig. 4a). Moreover, overexpression of SUPPRESSOR OF BIR1 (*SOBIR1)* leads to a constitutive activation of disease-resistance responses [58, 59]. Two transcripts homologs to SOBIR1 (Ach_contig37485; Ach_contig37484) were up-regulated only in ASM plants (Fig. 4a, Additional file 7: Table S4). Transcriptome analysis of other plant-pathogen interactions revealed that the SOBIR1 gene is transcriptionally regulated by biotic stress [15]. In *Sinapis alba* a SOBIR1 homologue was up-regulated after infection with *Alternaria brassicicola* [60]. Similarly, in *Malus domestica* a homologue of *At*SOBIR1 was up-regulated in plants resistant to *Erwinia amylovora* [61]. In the kiwifruit-Psa interaction, the SOBIR1 expression was related to acibenzolar-S-methyl application, suggesting a possible role for this gene in the SAR, as proposed for *Arabidopsis* [62].

Despite the high number of genes coding for PRR found in the *A. chinensis* var. *chinensis* genome [63] and the up-regulation of many of them at 3 hpi in untreated plants, this response is not sufficient to confer resistance to Psa. Thus, without acibenzolar-S-methyl treatment, kiwifruit is not capable of sustaining the activation of effective defensive mechanisms against the pathogen.

### Defense-related genes

Transcripts encoding several classes of chitinases (Ach_contig8637, Ach_contig3262, Ach_contig27724, Ach_contig24132, Ach_contig7693, Ach_contig25203), β-1,3-glucanase (Ach_contig27175), endochitinase (Ach_contig996, Ach_contig34595; Ach_contig6256) and *A. chinensis* thaumatin-like proteins (Ach_contig10838, Ach_contig19122) were up-regulated in ASM plants, indicating that these transcripts are related to the enhanced resistance response. The transcripts encoding PR1 homologs (Ach_contig12206, Ach_contig14525) were up-regulated in all infected samples with a higher expression in the ASM plants. The expression profile of Ach_contig14525 was confirmed by qRT-PCR (Additional file 5: Figure S2). Transcripts putatively encoding PR10 (Ach_contig15022; Ach_contig7880; Ach_contig18265; Ach_contig18266) were found up-regulated only in untreated plants at 3 hpi. OsPR10a and Pg1, PR10 orthologs, were up-regulated in response to exogenous ethylene treatment, in rice [64] and ginseng [65]. Moreover, two alfalfa PR10 genes, MsPR10.1A and MsPR10.1B, were responsive to ethylene and abscisic acid (ABA) treatment [65].

Actinidin 2d (Ach_contig26480; Ach_contig26479) is a member of the papain-like cysteine proteases family (PLCPs). Recent evidence indicates a key role for PLCPs in plant immunity [66, 67]. In the kiwifruit-Psa interaction the transcripts homologs to Actinidin showed a strong up-regulation in ASM plants with or without Psa inoculation. On the other hand, the same transcripts were down-regulated in untreated infected plants. This finding supports the idea that the up-regulation of transcripts coding for Actinidin enzymes is associated with the increased resistance of ASM plants to Psa. Indeed, it has been suggested that the presence of basic and acidic isoforms of Actinidin are reminiscent of PR proteins, having a role in defense against pathogens [68]. In addition, about 50 transcripts putatively annotated as disease resistance genes (homologues of TIR- and CC-NBS-LRR genes) were DEGs in one or more samples (Fig. 4b, Additional file 8: Table S5). Among them, three transcripts (Ach_contig24825; Ach_contig17717; Ach_contig17718) belonging to Resistance Gene Analogs RGA-like genes, and seven (Ach_contig18842; Ach_contig13980; Ach_contig35140; Ach_contig28816; Ach_contig35282; Ach_contig27985; Ach_contig35141) belonging to cysteine-rich like protein were induced in ASM plants upon inoculation.

### ROS detoxification related genes

**Fig. 5:**
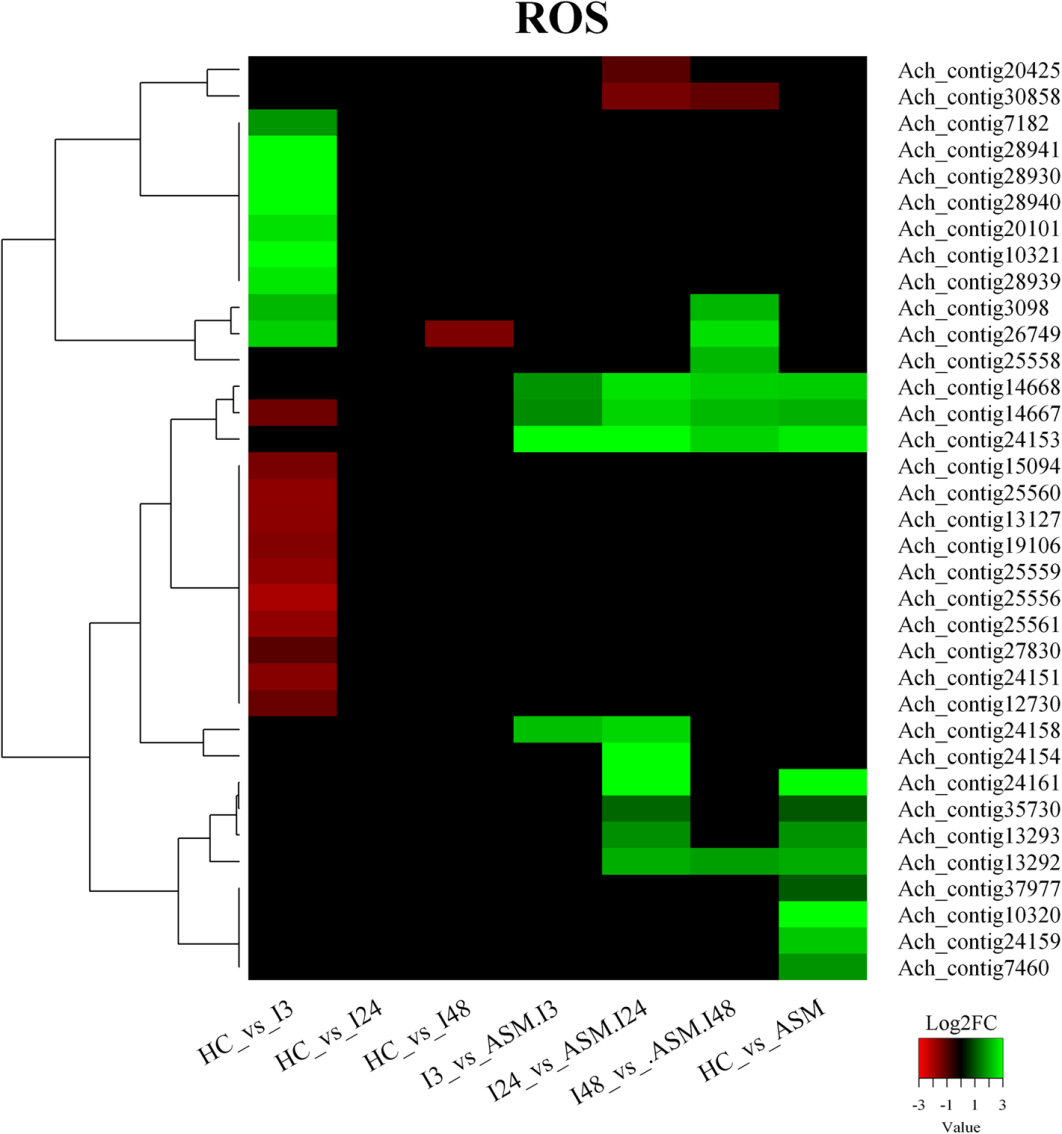
Heatmaps of Differentially Expressed Genes (DEGs) encoding putative Reactive Oxygen Species (ROS). Up-regulated transcripts (Log2FC ≥ 1) are in green, down-regulated transcripts (Log2FC ≤ − 1) are in red. Each row represents a transcript, each column a comparison. For the description of the gene names represented in the heatmaps refer to Additional file 9: Table S6

One of the earliest responses after pathogen recognition is the oxidative burst achieved through the production of ROS caused by nicotinamide adenine dinucleotide phosphate (NADPH) oxidases and the respiratory burst due to the oxidase homologues (RBOHs) multigenic family [69]. Accordingly, the transcriptomic analysis of the kiwifruit-Psa interaction has identified many DEGs involved in ROS production and detoxification. Four transcripts belonging to NADPH-oxidase (Ach_contig28941, Ach_contig28940, Ach_contig28939, Ach_contig28930) were up-regulated at 3 hpi in untreated plants, whereas only Ach_contig37977 was up-regulated in the acibenzolar-S-methyl-treated samples (Fig. 5). Moreover, antioxidant enzymes including superoxide dismutase (Ach_contig30858), catalase (Ach_contig25559, Ach_contig25558, Ach_contig25560, Ach_contig25556, Ach_contig25561), peroxidase (Ach_contig20101, Ach_contig10320, Ach_contig6123, Ach_contig27264, Ach_contig17103, Ach_contig18018, Ach_contig7182, Ach_contig18508, Ach_contig3949, Ach_contig35730, Ach_contig10321), ascorbate-oxidases (Ach_contig15094, Ach_contig26749), ascorbate peroxidase (Ach_contig12730, Ach_contig19106) and dehydroascorbate reductase (Ach_contig20425) were differentially expressed in several conditions (Fig. 5, Additional file 9: Table S6). Differential regulation of antioxidant enzymes, partially mediated by SA, may contribute to the increases of ROS and to the activation of defenses [70].

Hypersensitive Induced Response (HIR) proteins are a group of proteins involved in Hypersensitive Response [71]. Although there are no reports about *HIR* genes induced during SAR, the *PR-1* gene expression was elevated in transgenic *Arabidopsis* overexpressing the rice HR-induced gene *OsHIR-1* [72]. Interestingly, in ASM plants (both infected and not infected), a robust activation of transcripts encoding a protein related to HIR-1 (Ach_contig24151, Ach_contig27830, Ach_contig24154, Ach_contig24153, Ach_contig24161, Ach_contig24158) was found. Among them, Ach_contig24153 was used to confirm these data by qRT-PCR (Additional file 5: Figure S2). Besides, four transcripts homologous to peroxidase 4 (Ach_contig14667, Ach_contig14668, Ach_contig13292, Ach_contig13293) and belonging to the PR9 protein subfamily [73] were up-regulated in ASM plants regardless Psa inoculation. Members of the PR9 family are able to catalyze the synthesis of bioactive molecules that limit bacterial pathogen spreading through the establishment of physical barriers or the generation of toxic compounds, such as ROS [74].

### Photosynthesis-related genes

The infection strongly down-regulated photosynthetic related genes (Additional file 10: Table S7) as observed in other plant-pathogen interactions [75–78]. Moreover, in our experiment about 30 DEGs (Additional file 10: Table S7) were annotated as cytochrome P450 (CYP450). The cytochrome P450 (P450) superfamily is the largest family of plant metabolic enzymes and plant P450 families are highly divergent, reflecting diversification and neofunctionalization [79, 80]. CYP450s are involved in SA-dependent defense responses [81] such as lignin biosynthesis, callose deposition and cell wall reinforcement [82]. Nonetheless, P450 gene expression was not found to be consistently differentially expressed after acibenzolar-S-methyl treatment, or infection.

### Hormonal-pathway-related genes

**Fig. 6:**
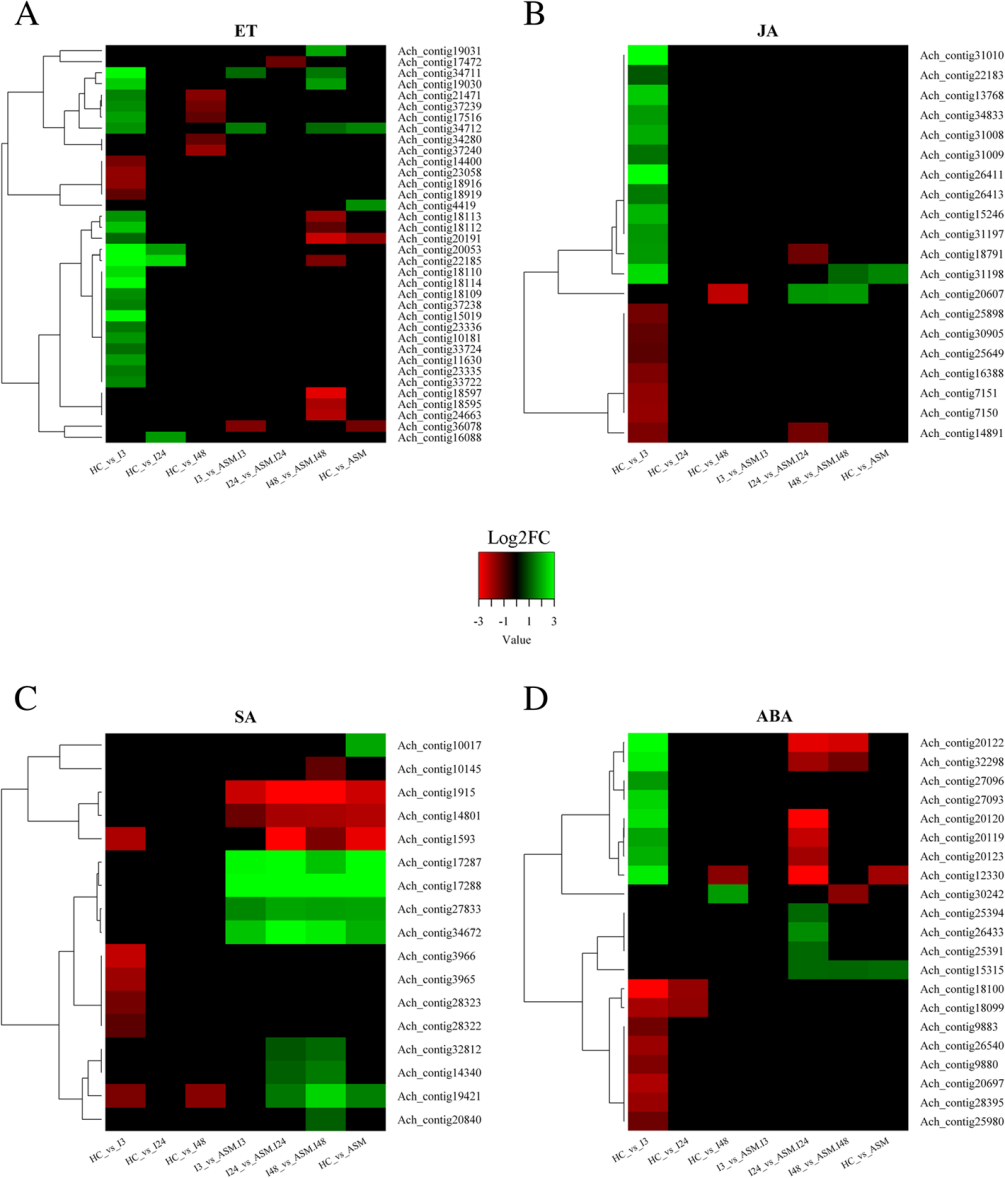
Heatmap describing the modulation of Differentially Expressed Genes (DEGs) involved in the hormonal pathways. **a** Ethylene (ET), **b** Jasmonic Acid (JA), **c** Salicylic Acid (SA) and **d** Abscisic acid (ABA). Up-regulated genes (Log2FC ≥ 1) are in green, whereas down-regulated ones (Log2FC ≤ − 1) are in red. The description of the genes represented in the heatmaps is reported in Additional file 11: Table S8, Additional file 12: Table S9, Additional file 13: Table S10, Additional file 14: Table S11, respectively

Several pathovars of *P. syringae* are known to produce compounds which manipulate the plant hormonal balance [83]. A well-known example of hormone manipulation by *P. syringae* involves the production of the phytotoxin coronatine (COR), which mimics JA functions. Other *P. syringae* pathovars, such as *P. syringae* pv. *tomato* (strain DC3000), modulate ABA level in order to suppresses stomata closure [84, 85]. Finally, some pathovars, such as *P. savastanoi* pv*. glycinea* and *P. savastanoi* pv. *phaseolicola* are capable of producing ET, which acts as a virulence factor by impacting ET production by the host [86]. Among the five biovars of Psa, strains of biovar 1 produce phaseolotoxin, a phytotoxin causing the halo blight disease, and strains of biovar 2 produce coronatine. Strains of biovar 3 do not produce any known toxin [7], and genes coding for ET biosynthesis, such as 2-oxoglutarate-dependent ethylene-forming enzyme (EFE), have not been found either. However, production of ethylene by some strains of Psa biovar 3 has been detected [12]. A subset of DEGs involved in ET biosynthesis and ET-signaling were found in kiwifruit plants upon Psa infection (Fig. 6a, Additional file 11: Table S8). The transcripts encoding homocysteine methyl transferase (HMT, Ach_contig26132, Ach_contig16088, Ach_contig22185), methionine synthase and S-adenosyl methionine-synthase (SAMS; Ach_contig18109, Ach_contig18113, Ach_contig17516, Ach_contig18112, Ach_contig18110, Ach_contig20053, Ach_contig18114) were up-regulated at 3 hpi in untreated plants only (Fig. 6a). The key enzymes of ET biosynthesis, *1-AMINO CYCLOPROPANE-1-CARBOXYLATE SYNTHASE* (*ACS*) and *ACC OXIDASE* (*ACO*), were also detected following Psa infection (Fig. 6a). Two transcripts encoding ACS enzymes were found significantly modulated only in control plants at 3 hpi: Ach_contig15019, encoding ACS2, was up-regulated, while Ach_contig23058, encoding ACS5, known to be unresponsive to exogenous ET treatment [87], was down-regulated.

Two transcripts homologous to *ENHANCED DISEASE RESISTANCE 1* (*EDR1*, Ach_contig3372; Ach_contig33724) were found up-regulated at 3 hpi in untreated plants (Fig. 6a). In *Arabidopsis*, *EDR1* negatively regulates SA-dependent defense responses, ABA signalling, and ET-induced senescence [88]. *EDR1* confers sensitivity to various pathogens such as *Erwinia cichoracearum* in cucurbits and *P. syringae* pv. *tomato* (strain DC3000) in tomato. *EDR1* is required for resistance to some hemibiotrophic/necrotrophic fungal pathogens through the induction of plant defensin (PDF), probably interfering with MYC2 function [89]. At 3 hpi in untreated plants, transcripts (Ach_contig10181) belonging to the PDF family were found to be up-regulated (Fig. 6a). Moreover, *Constitutive Triple Response1* (CTR1; Ach_contig33722, Ach_contig33724) and *Ethylene Insensitive2* (EIN2; Ach_contig23335, Ach_contig23336), which are key genes of the ET signaling pathway, were found to be up-regulated. At 3 hpi, in both untreated and ASM plants, transcripts encoding *EIN3 BINDING F-BOX1* (*EBF1*) and *EBF2* (Ach_contig34711, Ach_contig34712) were up-regulated. In *Arabidopsis*, these genes were up-regulated even in the absence of infection, and *EBF2* transcription was rapidly induced after exogenous application of ET [90].

The expression of ETHYLENE INSENSITIVE2 (EIN2) is supported by the activation of MYB44 [91] and, in turn, activates the expression of downstream transcription factors such as ETHYLENE RESPONSE FACTOR (AP2/ERF) [92]. Indeed, two MYB44 transcripts (Ach_contig27284 and Ach_contig27287) and about 25 transcripts encoding AP2/ERF transcription factors were up-regulated in response to the Psa infection in untreated plants (Fig. 6a).

Genes involved in JA-mediated responses were differentially expressed, mainly at 3 hpi in untreated plants (Fig. 6b, Additional file 12: Table S9). Transcripts corresponding to Allene Oxide Cyclase (AOC, Ach_contig13768) and OPC 8:0 CoA Ligase1 (OPCL1, Ach_contig13768) [93], two enzymes of the JA biosynthetic pathway, were all up-regulated. In addition, the expression of *JASMONATE-ZIM DOMAIN* (JAZ, Ach_contig31198, data confirmed by qRT-PCR, Additional file 5: Figure S2) was also found to be increased upon infection. JAZ proteins induce the expression of genes involved in the formation of the repressor complexes consisting of MYC2, a βHLH-type transcriptional regulator, NINJA adaptor-proteins and TOPLESS (TPL) co-repressors [94–96]. In ASM plants, none of these genes was modulated in relation to infection (Fig. 6b), thus suggesting that acibenzolar-S-methyl might minimize the hormonal imbalance caused by Psa to hijack plant defenses [12].

Few DEGs related to SA biosynthesis and signaling were detected in ASM plants regardless of Psa infection (Fig. 6c; Additional file 13: Table S10). SA biosynthesis occurs via the shikimic acid pathway, which involves the conversion of chorismate to isochorismate by *Isochorismate-synthase* (ICS) [91]. Control plants infected with Psa did not show modulation of ICS, while in acibenzolar-S-methyl-treated and infected plants, a transcript encoding ICS1 (Ach_contig10145) was found down-regulated at 24 hpi. In *Arabidopsis*, *ICS1* regulation is linked to calcium signaling through the transcriptional regulation of *Enhanced Disease Susceptibility-1* (EDS1) [97, 98]. In the same model plant, EDS1 works together with phytoalexin-deficient 4 (PAD4) to promote the hypersensitive response and SA accumulation [93]. In response to Psa, in kiwifruit, the transcript coding for EDS1-like lipase (Ach_contig27833) was up-regulated in all ASM samples. However, differently from *Arabidopsis*, PAD4 was not up-regulated in ASM kiwifruit plants. The up-regulation of PAD4 transcripts was instead detected in untreated infected plants at 3 hpi. Interestingly, in *Arabidopsis*, the up-regulation of PAD4 is essential for boosting ET production after pathogen infection [99–101]. Thus, the induction of PAD4 in kiwifruit untreated infected plants supports the role of ET as a positive regulator of host susceptibility in this pathosystem.

In ASM plants, both at 24 hpi and 48 hpi, transcripts coding for *Nonexpresser of PR proteins NPR1* and *NPR3* (Ach_contig14340 and Ach_contig32812, respectively) [102] were up-regulated. *NPR1* and *NPR3* are the main transcriptional regulators of the SA pathway [103]. The expression profile of Ach_contig14340 was confirmed by qRT-PCR (Additional file 5: Figure S2). The *Arabidopsis thaliana* NPR1-proteins act in concert with NIM1-INTERACTING 1 (NIMIN-1), NIMIN-2, and NIMIN-3 [104]. Accordingly, the transcripts encoding *NIMIN2* (Ach_contig17288; Ach_contig17287) were also induced in ASM plants (Fig. 6c; Additional file 13: Table S10): data confirmed by qRT-PCR (Additional file 5: Figure S2). These results confirm the priming effect of acibenzolar-S-methyl on SA-dependent defensive responses. Moreover, SA-dependent responses were activated by the infection only in ASM, thus corroborating the findings that SA mediates resistance against Psa in *A. chinensis* [12].

One of the first line of plant defenses against bacterial pathogen is the modulation of stomata closure [105]. SA induces stomatal closure in *Vicia faba* [106], *Phaseolus vulgaris* [107] and *Arabidopsis* [108] when applied on leaves. However, the effect of exogenous application of SA on stomatal movements may vary according to the plant species and the mode of application [109]. In this view, ABA plays a role in early plant defenses by inducing stomata closure upon recognition of pathogen-associated patterns [82, 110]. However, in post-infection phases, ABA generally exerts a negative role on plant immunity [111–113]. For example, *P. syringae* effector AvrPtoB stimulates ABA biosynthesis to weaken plant immune responses [78, 114]. ABA effect on stomata is mediated by cell turgor and *Ca*^*2+*^ balance in guard cells [115]. Indeed, *Ca*^*2+*^*-independent protein kinase SNF1 Related Kinase 2* (SNRK2) is a key regulator of the stomata closure [116]. In our experiment, two transcripts (Ach_contig20697 and Ach_contig15315) related with SNRK2 were identified as DEGs. Ach_contig20697 was down-regulated in untreated infected plants at 3 hpi, whereas Ach_contig15315 was up-regulated both in response to acibenzolar-S-methyl and to infection in treated samples at 24 hpi and 48 hpi (Fig. 6d; Additional file 14: Table S11).

In the absence of ABA, the PROTEIN PHOSPHATASE 2C (PP2C) acts as an inhibitor of the ABA signaling response by binding and blocking SNRK2 [117, 118]. In untreated plants at 3 hpi, several PP2Cs of class A [119], namely Ach_contig26540, Ach_contig9883, Ach_contig9880 and Ach_contig28395, were found to be down-regulated. Moreover, in the same plants at 3 hpi, four transcripts (Ach_contig20119, Ach_contig32298, Ach_contig20123, Ach_contig20122 and Ach_contig20120) encoding a class of intracellular ABA receptors (PYR/PYL/RCAR) [116] known to interact with PP2Cs [120], were also found to be up-regulated. In ASM plants at 24 hpi, the same transcripts were instead down-regulated (Fig. 6d). *Arabidopsis* plants are not able to close their stomata, even in the presence of ABA, if they are exposed to ET, confirming that ET represses ABA-dependent stomata closure [121]. In untreated plants at 3 hpi, a transcript related to *ABA repressor 1* (*ABR1*, Ach_contig18951, Fig. 8), which is a negative regulator of ABA signaling [122], was found to be up-regulated. *ABR1* upregulation was followed by down regulation at 24 hpi and 48 hpi (data confirmed by qRT-PCR; Additional file 5: Figure S2). Furthermore, in the same plants and at the same time points, transcripts encoding for the E3 ligase ARM REPEAT PROTEIN INTERACTING with ABF2-like (*ARIA-like TFs*; Ach_contig27093, Ach_contig27096) were found to be up-regulated. ARIA-like TFs were involved in the ubiquitination of ABF2, which controls ABA-responsive gene expression [123]. Finally, in ASM plants at 24 hpi, the bZIP transcription factor ABI5 (Ach_contig26433, Ach_contig25391, Ach_contig25394), known to be transcriptionally induced by ABA [124, 125], was up-regulated (Fig. 6d).

All these findings suggest a possible role of ET as a Psa virulence factor concurring in stomata opening by repressing ABA-mediated signaling.

### Transcription factors

**Fig. 7:**
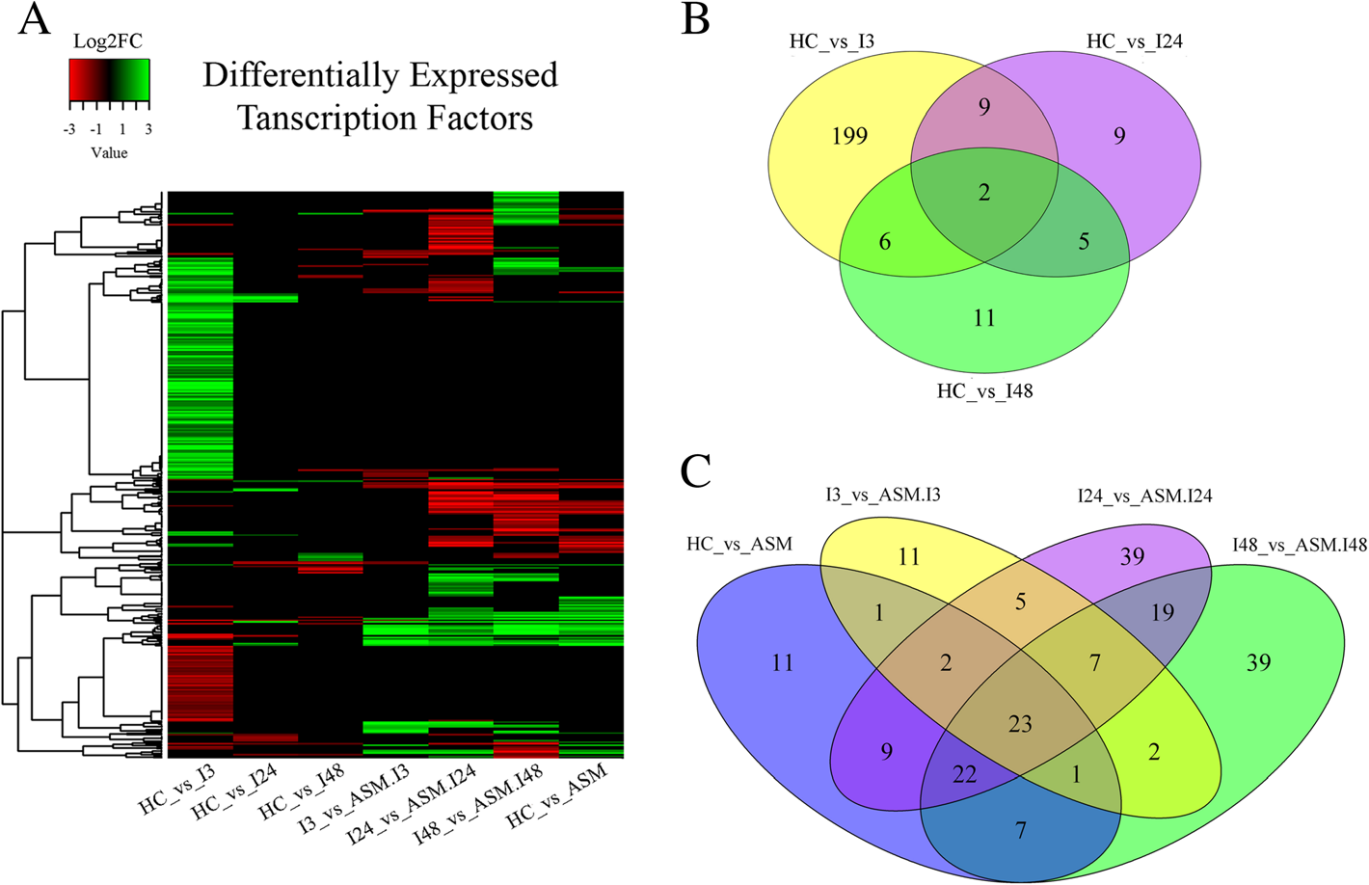
Panel **a**: Heatmap showing differentially expressed transcription factors (TFs). Each row represents a transcript; each column represents a library comparison. A dendrogram of the correlation among transcripts is shown on the left of the heatmap. The green color represents up-regulated genes (Log2FC ≥ 1) and red color represents down-regulated genes (Log2FC ≤ − 1). The description of the genes represented in the heatmap is reported in Additional file 16: Table S12; Panel **b**: Venn diagram showing the overlapping of differentially expressed TFs modulated in infected plants at 3, 24 and 48 hpi. Panel **c**: Venn diagram showing the overlapping of TFs modulated in acibenzolar-S-methyl treated plants inoculated with Psa at 3, 24 and 48 hpi

Transcription factors (TFs) play a key role in the regulation of plant responses to pathogens. In the kiwifruit transcriptome, 3009 transcripts belonging to 53 families of TFs and to 13 families of Chromatin Remodeling Factors were detected (Additional file 15: Figure S4; Additional file 16: Table S12). Concerning TFs, 367 transcripts were DEGs in at least in one condition (Fig. 7a, Additional file 17: Table S13). A total of 216 TFs were modulated at 3 hpi in untreated plants. Out of these, 199 TFs were modulated only in this condition (Fig. 7b) with the most represented families corresponding to APETALA2/ETHYLENE RESPONSIVE FACTOR (AP2/ERF) and WRKY. In ASM plants, 187 transcripts coding for TFs were modulated, with 11 identified either in ASM plants without Psa or in untreated plants at 3 hpi. Finally, 39 TFs were exclusively modulated at either at 24 or 48 hpi (Fig. 7c).

**Fig. 8:**
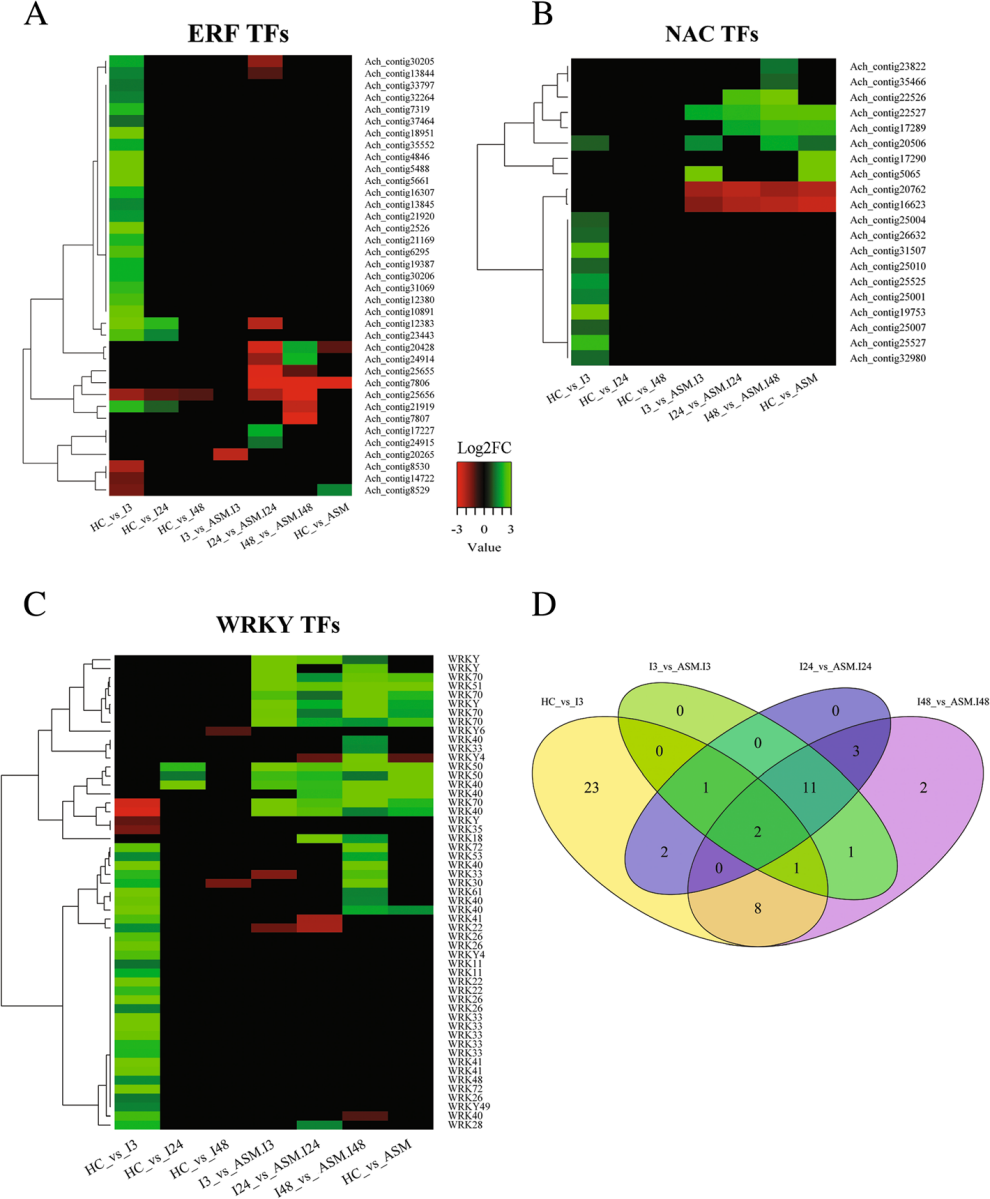
Heatmaps showing transcription factors (TFs) differentially expressed in the kiwifruit-Psa interaction. Panel **a**: Ethylene responsive Factors (ERFs); Panel **b**: NAC TFs; Panel **c**: WRKY TFs. The green color represents up-regulated genes (Log2FC ≥ 1) and red color represents down-regulated genes (Log2FC ≤ − 1). Panel **d**: Four-ways Venn diagram showing WRKY TFs comparison between early stages of Psa inoculation in kiwifruit untreated plants against acibenzolar-S-methyl treated ones. For the description of the gene names represented in the heatmap refer to Additional file 18: Table S14, Additional file 19: Table S15 and Additional file 20: Table S16, respectively

AP2/ERF is a large family of plant TFs divided into three subfamilies: AP2, RAV and ERF [126]. Individual members of the ERF family have been shown to be either positive or negative regulators of the defense response. A total of 35 AP2/ERF were modulated in the kiwifruit-Psa interaction (Fig. 8a). Among them, 23 AP2/ERF, mainly related to ERF subfamily IX [127], were up-regulated in untreated plants at 3 hpi, and only three of them were also down-regulated in ASM samples inoculated with Psa (Fig. 8a, Additional file 18: Table S14).

TFs belonging to ERF subfamily IX have been often linked with response to pathogen infection in *Arabidopsis*, rice and grape [126, 128]. In detail, in *Arabidopsis*, ERF1, belonging to subfamily IX, is involved in both JA and ET signaling. ERF1 over-expression increases resistance to necrotrophic fungi and enhances susceptibility to the hemibiotroph *P. syringae* pv*. tomato* [53]. Moreover, Ach_contig21919; Ach_contig30206; Ach_contig30205; Ach_contig21920; Ach_contig13845; Ach_contig13844, belonging to family IX, were annotated as *AdERF11*, *AdERF12* and *AdERF13.* These ERF TFs of *Actinidia deliciosa* are known to control the expression of other ERF genes in response to ET and JA [127].

Two transcripts (Ach_contig5488; Ach_contig19387) belonging to subfamily VIII were up-regulated at 3 hpi in untreated plants (Additional file 16: Table S12). Interestingly, the expression of genes of subfamily VIII can be rapidly induced by ET and JA synergistically [128, 129]. Likewise, three transcripts (Ach_contig12383; Ach_contig23443; Ach_contig12380), up-regulated at 3 hpi in untreated plants, were identified as *AdERF4* that, along with *AdERF5* (Ach_contig33797), belongs to subfamily VII (Additional file 18: Table S14). The members of this subfamily have been associated with the modulation of ET during hypoxia and with grape resistance to the necrotrophic pathogen *Botrytis cinerea* [130]. Finally, the transcript corresponding to ABR1-like, belonging to Subfamily X*,* was one of the most up-regulated genes as reported in section “Hormonal Balance”.

Plants treated with acibenzolar-S-methyl showed a general down-regulation of ERF genes with the exception of transcript Ach_contig8529 which is an ortholog of Tobacco stress-induced gene 1 (*Tsi1*) (Fig. 8a). *Tsi1,* which exhibits characteristic features of group VI, is known to be responsive to SA treatment [131]. Moreover, the over-expression of *Tsi1* in tobacco and pepper increased resistance to viral, bacterial and oomycete pathogens [131, 132]. Therefore, *Tsi1* could be one of the main candidate genes involved in increased defenses against Psa in ASM kiwifruit plants.

Additionally, to AP2/ERF-related transcripts, 20 contigs belonging to the NAC TF family were identified (Fig. 8b). The NAC TF family, which include three subfamilies, namely NAM, ATAF and CUC, regulates a variety of plant processes, including the response to biotic and abiotic stresses [133, 134]. Within this family, in *Arabidopsis*, ATAF1/NAC2 is known to be a negative regulator of ABA signalling and is related to ET signaling [135, 136]. In our experiments, in untreated infected plants at 3 hpi, an up-regulation of transcripts homologous to ATAF1/NAC2 (Ach_contig25527; Ach_contig25525) was recorded. Moreover, others transcripts (Ach_contig26632; Ach_contig19753; Ach_contig31507; Ach_contig32980; Ach_contig25010; Ach_contig25004; Ach_contig25007), homologous to NAC TFs, were also up-regulated (Fig. 8b, Additional file 19: Table S15).

Acibenzolar-S-methyl modulated a number of NAC TFs (Fig. 8b). Some NAC TFs are positive regulators of SA-mediated responses, such-as NAC090 [137] whose transcript (Ach_contig20506) was up-regulated in all ASM samples. The transcript Ach_contig20762, a homolog to ANAC100 (*Arabidopsis* NAC domain containing protein 100), was down-regulated in all ASM samples. Close homologs of ANAC100 are involved in ET signaling and biotic stress response [138]. Therefore, the down-regulation of this transcript in ASM plants may indicate a regulatory SA-dependent mechanism.

Finally, acibenzolar-S-methyl treatments also up-regulated a transcript homologous to ANAC042, a TF involved in the induction of camalexin biosynthesis, in *Arabidopsis*. Camalexin, a phytoalexin, is accumulated in response to pathogen infection through complex signaling networks, including SA-, JA-, and ET-dependent pathways, as well as glutathione status and ROS generation [139]. Phytoalexins are known to have an antimicrobial activity [140, 141].

A total of 53 WRKY TFs [142] were listed as DEGs, and among them, 23 were exclusively modulated in untreated plants at 3 hpi (Fig. 8c and d), highlighting the role of these TFs in the transcriptional reprogramming of the kiwifruit plant during Psa infection (Fig. 8c, Additional file 20: Table S16). Transcripts Ach_contig37648, Ach_contig20598, Ach_contig20597, Ach_contig37652, Ach_contig15612 and Ach_contig17244, related to the rapidly pathogen induced AtWRKY33, were up-regulated in untreated plants at 3 hpi. AtWRKY33 acts as a positive regulator of resistance toward the necrotrophic fungi *Alternaria brassicicola* and *Botrytis cinerea* [143]. In addition, its expression does not require SA-dependent signaling, but it is dependent on PAD4 [144, 145].

After *P. syringae* infection in *Arabidopsis*, WRKY33 is released in the nucleus where it induces JA\ET-related defense genes, repressing at the same time the genes related to SA-dependent defense [146]. Ach_contig20597, belonging to WRKY33, was used to confirm these data by qRT-PCR (Additional file 5: Figure S2). Moreover, WRKY33 and WRKY22 are known to activate the PAMP signaling cascade through MAPK cascade signal transduction pathways. WRKY22 activation is driven by the MAPK3/MAPK6 signal cascade and requires the cooperation of other WRKY TFs for the induction of resistance to bacterial and fungal pathogens [147]. In our experiments, Ach_contig16581 and Ach_contig14586 were annotated as WRKY22. In addition, Ach_contig22394, Ach_contig26220 Ach_contig26219, annotated as MAPK3 and MAPK6 related to MAPK3/MAPK6, were up-regulated at 3 hpi in untreated plants (Additional file 21: Table S17).

AtWRKY18 is closely related to AtWRKY40 and AtWRKY60. These three WRKY TFs interact in a complex manner to regulate plant defensive responses [148]. A transcript related to AtWRKY18 (Ach_contig11697) was identified as a DEG in ASM plants at 24 hpi and 48 hpi. Moreover, eight transcripts putatively encoding AtWRKY40 (Ach_contig15900; Ach_contig15903; Ach_contig6471; Ach_contig6470; Ach_contig7044; Ach_contig5585; Ach_contig10300; Ach_contig19510) were up-regulated at several time points following Psa inoculation in both control and ASM plants. In transgenic *Arabidopsis*, where AtWRKY18 was constitutively expressed, an increased resistance to *P. syringae* was observed. On the other hand, its co-expression with WRKY40 and WRKY60 has a redundant function and negatively regulates the resistance to *P. syringae* [149].

The acibenzolar-S-methyl treatment acts on a class of WRKY TFs more related to SAR response, modulating distinct transcripts from those observed in infected untreated plants. In detail, five transcripts (Ach_contig20526; Ach_contig20525; Ach_contig22553; Ach_contig22554; Ach_contig16016), related to AtWRKY70 (Additional file 5: Figure S2 and Additional file 20: Table S16), were reported as DEGs and up-regulated specifically after acibenzolar-S-methyl treatment (Fig. 8c). Among them, Ach_contig20526 was used to confirm these data by qRT-PCR (Additional file 5: Figure S2). AtWRKY70, similarly to WRKY51 and WRKY50, acts as a positive regulator of SA-dependent responses and as a negative regulator of JA-dependent responses [150, 151]. Moreover, AtWRKY70, together with its closely related AtWRKY53, plays a key role in the regulation of SAR and innate immunity in *Arabidopsis* [137]. Recent evidence suggests that AtWRKY70, and other WRKY TFs, are the targets of *Nonexpresser of PR proteins* NPR1, a key regulator of SA-dependent defenses and SAR [152]. In the kiwifruit plant-Psa interaction, three transcripts (Ach_contig16947; Ach_contig20013; Ach_contig20015), related to WRKY50 and WRKY51, were observed as DEGs and their expressions were preferentially linked to acibenzolar-S-methyl treatment (Fig. 8c). In this view, our results suggest that WRKY TF may be involved in the defense response to Psa and may be associated with ET and JA signaling in kiwifruit plants.

### Network analysis WGCNA

Another perspective on this transcriptomic analysis was given by weighted gene co-expression network analysis (WGCNA). WGCNA allows to get a better understanding of which genes within this plant-pathogen interaction signalling networks, were the most connected hubs. Twenty-one modules were detected, assigned colour names and correlated to Psa inoculation and ASM treatment effects over the time points.

The kME (module eigengene-based connectivity) measure was calculated for each gene to every twenty-one modules with the score ranging between 1 (perfectly positively correlated) to − 1 (perfectly negatively correlated). kME scores were computed for each module in order to detect genes which can act as a hub in more than one network. The most interesting module detected in our analysis was “darkorange2” which is highly correlated (0.98; Pval 5e-12) with the ASM treatment (Additional file 22: Figure S5). Moreover, “darkorange2” shown positive correlation along the time course of inoculated ASM plants but negative correlation with Psa inoculated untreated plants.

The top hub of “darkorange2” network was an inactive leucine-rich repeat receptor-like protein kinase; several other leucine-rich repeat receptor-like were detected in this module indicating the importance of these classes of genes in the defense response [153]. About 222 hubs transcripts involved in the defense response with a kME score greater than 0.90 were detected in this module (Additional file 23: Table S18). About 179 of them were identified as DEGs in several of the comparisons discussed above. Among them, Ach_contig19122, which encoded thaumatin-like protein, was differentially expressed in ASM plants along the time course and was positively correlated with “darkorange2”. In contrast, Ach_contig19122 was negatively correlated with the “skyblue2” network which represents a module preferentially associated with the Psa infection in untreated plants (Additional file 22: Figure S5). Also Ach_contig26479, encoding actinidin act2d, and Ach_contig7693, encoding a class I chitinase, corroborated this observation (Additional file 23: Table S18). WGCNA also confirmed a clear separation by treatment and infection (Additional file 22: Figure S5).

## Conclusions

In this study, we characterized the transcriptome of *A. chinensis* var. *chinensis* and identified genes differentially expressed after infection with Psa in plants treated and not treated with acibenzolar-S-methyl. Inoculation by Psa led to a large plant response in the initial phase with 1596 genes being over expressed and 1152 being repressed at 3 hpi. Nevertheless, this response was much reduced at 24 hpi and 48 hpi when the number of DEGs was only 272 and 341 respectively. Thus, while endophytic populations of Psa increased rapidly, the plants’ reactions were relatively muted. It is interesting to speculate whether this limited reaction to Psa infection is a consequence or a cause of Psa invading the plant tissues.

Some clues for answering this question might be found comparing gene expression in plants treated or not treated with acibenzolar-S-methyl prior to inoculation. In non-infected plants acibenzolar-S-methyl modulated the expression of 475 genes including an up-regulation of several PRRs, defense-related genes (e.g. NBS-LRR genes and Actinidin) and genes involved in the SA pathway (e.g. NIMIN2 and EDS1). The expression of genes involved in the JA/ET pathways was mostly unchanged: these results were expected since acibenzolar-S-methyl is known to elicit the SA pathway. In ASM plants the initial reaction (3 hpi) was not as pronounced as in the acibenzolar-S-methyl-untreated ones but it grew stronger with time (from 510 DEGs at 3 hpi to 1374 and 1252 DEGs at 24 hpi and 48 hpi, respectively). Notwithstanding, at 24 hpi or 48 hpi the DEGs in the ASM plants were largely different from those found in the acibenzolar-S-methyl-untreated ones with only 46 DEGs in common between acibenzolar-S-methyl-untreated (24 hpi or 48 hpi) and -treated without inoculation. A larger overlap was found in ASM plants before and after inoculation (acibenzolar-S-methyl-treated vs. -treated and inoculated at 24 hpi or 48 hpi 282 DEGs). Among the genes induced by acibenzolar-S-methyl, we found PRR genes and those involved in ROS detoxification (e.g. catalase and superoxide dismutase) or in the SA elicitation pathway (e.g. EDR1 and AP2/ERF). In addition, none of the genes involved in JA (e.g. AOC, JAZ proteins) or ET (e.g. EDR1, ACS3, SAMS, HMT) elicitation pathways that were up-regulated at 3 hpi in acibenzolar-S-methyl-untreated plants, were found to be DEGs in acibenzolar-S-methyl-treated samples.

A number of the genes up-regulated after acibenzolar-S-methyl treatment, that were involved in or contributed to the SA elicitation pathway, were further up-regulated after inoculation (e.g. EDS1, NIMIN2). A similar profile was found for several TFs such as WRKY40 and WRKY70. These findings suggest that the expression of these genes is primed by the acibenzolar-S-methyl treatment.

Genes involved in the ET pathway (e.g. AdERF11–14, HMT and SAMS) as well as genes involved in the JA pathway (e.g. AOC and OPCL1) were up-regulated in acibenzolar-S-methyl-untreated plants at 3 hpi. Since the JA and the ET pathways are antagonistic to the SA pathway, which is known to limit Psa infection [12], the induction of SA by acibenzolar-S-methyl treatment might contribute to the resistant phenotype. Inoculation of untreated plants also leads to increased expression of ERF genes also involved in the ET pathway. In contrast, inoculation of ASM plants leads to over-expression of genes involved in the SA pathway but not those involved in the ET or JA pathway.

The consistent picture emerging from this study suggests that the host response is partly dictated by the pathogen, which reduces the defense capacity of the plant, as long as within 24 hpi the affected cells are unable to prevent bacterial multiplication and therefore disease. In contrast, in ASM plants a different molecular response primed by acibenzolar-S-methyl treatment blocks Psa multiplication, thus preventing the disease symptoms. Moreover, these results elucidate and confirm the mechanisms of Psa control strategies in open field based on acibenzolar-S-methyl.

Some cultivars of *A. chinensis* var. *deliciosa*, e.g. ‘Hayward’, are reported to show a stronger reaction to acibenzolar-S-methyl treatment than the cultivar used in this study [12]; it would be interesting to compare gene expression of such cultivar with that presented in this study.

This study not only gave us a better understanding of the early interaction between Psa and its host, but it also indicates new avenues for the selection of novel elicitors and selection of kiwifruit genotypes which will respond well to those elicitors or selection of kiwifruit genotypes which will not respond to Psa manipulation.

## Additional files

Additional file 1: **Table S1.** List of primers used for qRT-PCR of selected differentially expressed genes (DEGs). (XLSX 13 kb)

Additional file 2: **Table S2.** Summary of annotations of the *Actinidia chinensis* var. *chinensis* transcriptome. (DOC 66 kb)

Additional file 3: **Figure S1.** Main characteristics of the annotation of the *Actinidia chinensis* var. *chinensis* reference transcriptome. Panel **A**: contig distribution by length. Panel **B**: contigs distribution by E-value. Panel **C**: contig distribution by similarity. Panel **D** The contigs of the *A. chinensis* var. *chinensis* reference transcriptome have been classified in Clusters of Orthologous Groups (COGs) functional annotations and organized into 24 function categories. Panel **E** Most relevant functional categories of the Groups of Orthologs obtained with the *A. chinensis* var. *chinensis* transcriptome. Venn diagrams illustrating the distribution of similarity search results made with the *A. chinensis* var. *chinensis* transcriptome. In panel **F,** the BLAST results against NR protein, NR nucleotide, RefSeq and SwissProt databases are reported. The total number of annotations obtained from these databases was 29,583 (74.73% of total contigs). In panel **G** are shown the annotation results obtained from COGs, InterProScan and KEGG. The number of contigs showing a hit against these databases was 26,353 (66.57% of total contigs). Panel **H** reports the intersection among the annotations presented in A and B with the results obtained from the BLASTx search against the Kiwifruit Genome protein database. Summarizing the results obtained from all the queried databases, a total of 34,039 contigs (85.99% of total contigs) were annotated. (TIF 2577 kb)

Additional file 4: **Table S3.** List of all differentially expressed genes (DEGs) of *Actinidia chinensis* var. *chinensis* modulated in response to *Pseudomonas syringae* pv. *actinidiae* inoculation both in acibenzolar-S-methyl treated and untreated plants. (XLSX 1256 kb)

Additional file 5: **Figure S2.** qRT-PCR analysis was employed to validate the expression of twenty differentially expressed genes (DEGs). Ach_contigs and primers used are reported in Additional file: Table ST1, the PCR conditions are described in Material and Methods. Gene expression expressed as fold change and time course is indicated in the X axis. Standard errors are indicated. Results of quantitative PCRs were in agreement with RNA-seq experiment. (TIF 1088 kb)

Additional file 6: **Figure S3.** The MapMan figure of the “Biotic stress” was obtained by running the Mercator tool (http://mapman.gabipd.org/web/guest/mercator) with default parameters to assign MapMan bins to *Actinidia chinensis* var. *chinensis* transcripts. (TIF 3354 kb)

Additional file 7: **Table S4.** List of subset of differentially expressed genes (DEGs) related with PRRs genes. (XLSX 505 kb)

Additional file 8: **Table S5.** List of subset of differentially expressed genes (DEGs) related with defense genes. (XLSX 484 kb)

Additional file 9: **Table S6.** List of subset of differentially expressed genes (DEGs) related to ROS detoxification. (XLSX 470 kb)

Additional file 10: **Table S7.** List of subset of differentially expressed genes (DEGs) related to photosynthesis. (XLSX 43 kb)

Additional file 11: **Table S8.** List of subset of differentially expressed genes (DEGs) involved in ethylene biosynthesis and signalling. (XLSX 467 kb)

Additional file 12: **Table S9.** List of subset of differentially expressed genes (DEGs) involved in jasmonic acid-mediated response. (XLSX 462 kb)

Additional file 13: **Table S10.** List of subset of differentially expressed genes (DEGs) involved in salicylic acid biosynthesis and signalling. (XLSX 464 kb)

Additional file 14: **Table S11.** List of subset of differentially expressed genes (DEGs) involved in abscisic acid biosynthesis and signalling. (XLSX 463 kb)

Additional file 15: **Figure S4.** Distribution of transcription factors (TFs) in *Actinidia chinensis* var. *chinensis* transcriptome based on BLASTx against plantTFDBcat (http://plantgrn.noble.org/PlantTFcat). Chromatin remodelling factors and families of TF with less than 5 transcripts are not shown. C2C2 family harbours: CO-like Dof, GATA, LSD and YABBY TFs. AP2/ERF includes the AP2, ERF and RAV classes of TFs. ARF and B3 classes belong to B3 superfamily of TFs. HD-ZIP, TALE, WOX, HB-PHD, and HB-other were grouped in the HB class. GARB family of TFs consists of ARR and G2-like classes. Others category consists of FHA-SMAD, GAGA-Binding-like, GeBP, HSF-type-DNA-binding, Nin-like, PAZ-Argonaute, PLATZ, Znf-B, Znf-LSD, SAP, SBP, TCP, TUBBY, FAR, CG1-CAMTA, E2F-DP, STY-LRP1, CW-Zn. Numbers of transcripts for each family were summarized in the figure and are detailed in Additional file: Table S12. (TIF 750 kb)

Additional file 16: **Table S12.** Distribution of transcription factors (TFs) in *Actinidia chinensis* var. *chinensis* transcriptome. (XLSX 357 kb)

Additional file 17: **Table S13.** List of subset of differentially expressed genes (DEGs) related to transcription factors. (XLSX 545 kb)

Additional file 18: **Table S14.** List of subset of differentially expressed genes (DEGs) related to AP2/ERF transcription factors. (XLSX 463 kb)

Additional file 19: **Table S15.** List of subset of differentially expressed genes (DEGs) related to NAC transcription factors. (XLSX 458 kb)

Additional file 20: **Table S16.** List of subset of differentially expressed genes (DEGs) related to WRKY transcription factors. (XLSX 463 kb)

Additional file 21: **Table S17.** List of subset of differentially expressed genes (DEGs) related to MAPK cascade signal transduction. (XLSX 458 kb)

Additional file 22: **Figure S5.** Heatmap of the correlation of WGCNA modules with traits (ASM treatment and Psa inoculation). 21 modules were detected and named with colour names. The grey category is not a real module: it collect all the leftover genes not enough correlated with one of the other significant coloured modules. In each square the upper value is kME (module eigengene-based connectivity) while the lower value is the *P*-value of the correlation. (TIF 1752 kb)

Additional file 23: **Table S18.** List of the genes obtained by WGCNA and the kMEs for each module. (XLSX 9185 kb)

## Abbreviations

ABA: Abscisic acid
ABI: Abscisic acid insensitive
ABRE: ABA-responsive element
ACO: 1-amino cyclopropane-1-carboxylate oxidase
AOS: Allene oxide synthase
AP2/ERF: APETALA 2/ethylene-responsive element binding factor
ASM: Plants treated with acibenzolar-S-methyl not inoculated with Psa
ASM.I3, ASM.I24, ASM.I48: Plants treated with acibenzolar-S-methyl and then inoculated with Psa at 3, 24, 48 h post inoculation
CaM: Calmodulin
CLSM: Confocal laser scanning micrographs
COI: Coronatine insensitive
DEGs: Differentially expressed genes
EDS: Enhanced disease susceptibility
EFE: 2-oxoglutarate-dependent ethylene-forming enzyme
EIL: Ethylene insensitive like
EIN: Ethylene insensitive
ET: Ethylene
ETI: Effector-triggered immunity
FDR: False discovery rate
FMO1: Flavin-contain monooxigenase1
HC: Healthy Control plants
hpi: Hour post inoculation
HR: Hypersensitive response
I3, I24, I48: Inoculated plants, 3, 24, 48 h post-inoculation
JA: Jasmonic acid
JAZ: Jasmonate-zim domain
KEGG: Kyoto Encyclopedia of Genes and Genomes
kME: Module eigengene-based connectivity
log2fc: Log2 fold change
LOX: Lipoxygenase
LRR: Leucine rich-repeat
LRR-RLKs: Leucine-rich repeat receptor-like protein kinases
MAMP/PAMP: Microbe/pathogen-associated molecular pattern
MAPK: Mitogen-activated protein kinase
MeJA: Methyl jasmonic acid
NAC: No apical meristem, ATAF1/2, CUP-SHAPED COTYLEDON 2
NBS-LRR: Nucleotide-binding site leucine-rich repeat
NCED: 9-cis-epoxycarotenoid dioxygenase
NPR: Nonexpresser of PR genes
PAD: Phytoalexin deficient
PDF: Plant defensin
PP2C: Type 2C protein phosphatase
PR: Pathogenesis related
Psa: *Pseudomonas syringae* pv*. actinidiae*
PTI: PAMP-triggered immunity
PYR/PYL/RCAR: Pyrabactin resistance/PY- like/regulatory component of ABA receptor
qRT-PCR: quantitative reverse transcription PCR
R-genes: Resistance genes
RLK: Receptor Like Kinases
RNAseq: RNA-Sequencing
ROS: Reactive oxygen species
SA: Salicylic acid
SAR: Systemic acquired resistance
SnRK: SNF1-related protein kinase
TFs: Transcription factors
TLPs: Thaumatin-like proteins
WGCNA: Weighted gene coexpression network analysis
WRKY: Transcription factor containing a highly conserved WRKY domain
