# Supplementary material for: Comparative transcriptome analysis of the interaction between Actinidia chinensis var. chinensis and Pseudomonas syringae pv. actinidiae in absence and presence of acibenzolar-S-methyl
Source: BMC Genomics. 2018 Aug 6;19:585. doi: 10.1186/s12864-018-4967-4 (PMC6090863; doi:10.1186/s12864-018-4967-4)
Supplement: Supplementary file 2 — Table S2. Summary of annotations of the Actinidia chinensis var. chinensis transcriptome. (DOC 66 kb) [file 12864_2018_4967_MOESM2_ESM.doc]

**Additional file 2: Table S2** Summary of annotations of the *Actinidia chinensis var chinensis* transciptome

**A.** Summary of *Actinidia chinensis var chinensis* contigs.

|  | ***N° contig*** | ***N° bases (Mbp)*** | ***N50 (bp)*** | ***Shortest (bp)*** | ***Longest (bp)*** | ***Mean (bp)*** | ***Median (bp)*** |
| --- | --- | --- | --- | --- | --- | --- | --- |
| Initial Contigs | 63943 | 68.93 | 1614 | 201 | 11870 | 1078 | 812 |
| Final Contigs | 39607 | 39.97 | 1472 | 201 | 11870 | 933 | 619 |

**B**. Summary of annotations of the *Actinidia chinensis var chinensis* transciptome.

|  | ***Sequences (n)*** | ***Sequences (%)*** | ***Annotations (n)*** | ***Functional classification*** |
| --- | --- | --- | --- | --- |
| Contigs | 39,607 | 100 | - | - |
| Contig hits against NR protein database | 28,812 | 72.74 | 28,812 | 21,995 accessions |
| Contig hits against RefSeq protein database | 28,657 | 72.35 | 28,657 | 20,305 accessions |
| Contig hits against Swiss-Prot database | 20,974 | 52.96 | 20,974 | 10,399 accessions |
| Contig hits against NR nucleotide database | 17,422 | 43.84 | 17,422 | 13,609 accessions |
| Contig hits against COG database | 12,215 | 30.84 | 12,215 | 5209 accessions  24 categories |
| Contig hits against InterPro database | 16,073 | 40.58 | 38,763 | 5279 domains/families |
| Contig hits against other domain/family databases | 23,250 | 58.7 | 53,993 | 7021 domains/families |
| GO Slim Plant annotations for NR protein hits | 21,645 | 54.65 | 124,931 | 3 main categories  101 sub-categories |
| GO Slim Plant annotations for RefSeq protein hits | 20,149 | 50.87 | 110,732 | 3 main categories  100 sub-categories |
| GO Slim Plant annotations for Swiss-Prot hits | 18450 | 46.58 | 202,606 | 3 main categories  102 sub-categories |
| KEGG Annotations | 5,125 | 12.94 | 5,125 | 2,853 accessions |
| All annotated contigs | 32,610 | 82.33 | - | - |

**C** The twenty most abundant Interpro domains/families in *A. chinensis var chinensis* transcriptome.

| ***Conserved domain family*** | ***Accession_ID*** | ***Feature*** | ***Sequence (n)*** |
| --- | --- | --- | --- |
| Protein kinase-like domain | IPR011009 | Domain | 819 |
| Protein kinase, catalytic domain | IPR000719 | Domain | 761 |
| P-loop containing nucleoside triphosphate hydrolase | IPR027417 | Domain | 703 |
| Serine/threonine- / dual specificity protein kinase, catalytic domain | IPR002290 | Domain | 522 |
| Tyrosine-protein kinase, catalytic domain | IPR020635 | Domain | 481 |
| Serine/threonine-protein kinase, active site | IPR008271 | Active_site | 444 |
| Zinc finger, RING/FYVE/PHD-type | IPR013083 | Domain | 404 |
| Protein kinase, ATP binding site | IPR017441 | Binding_site | 389 |
| Pentatricopeptide repeat | IPR002885 | Repeat | 367 |
| Nucleotide-binding, alpha-beta plait | IPR012677 | Domain | 288 |
| NAD(P)-binding domain | IPR016040 | Domain | 284 |
| Zinc finger, RING-type | IPR001841 | Domain | 262 |
| RNA recognition motif domain | IPR000504 | Domain | 261 |
| Concanavalin A-like lectin/glucanase, subgroup | IPR013320 | Domain | 247 |
| Homeodomain-like | IPR009057 | Domain | 246 |
| Serine-threonine/tyrosine-protein kinase catalytic domain | IPR001245 | Domain | 239 |
| Tetratricopeptide-like helical | IPR011990 | Domain | 235 |
| Armadillo-type fold | IPR016024 | Domain | 231 |
| WD40/YVTN repeat-like-containing domain | IPR015943 | Domain | 230 |
| Leucine-rich repeat | IPR001611 | Repeat | 227 |

**D** Summary of annotation against other domain/family databases.

|  | ***Sequences (n)*** | ***Sequences (%)*** | ***Annotations (n)*** | ***Functional classification*** |
| --- | --- | --- | --- | --- |
| Contig hits against GENE3D | 11,074 | 27.96 | 14,008 | 990 |
| Contig hits against PROMOD | 202 | 0.51 | 205 | 90 |
| Contig hits against PFAM | 14,948 | 37.74 | 18,879 | 3256 |
| Contig hits against SUPERFAMILY | 11,195 | 28.27 | 13,162 | 830 |
| Contig hits against PANTHER | 16,389 | 41.38 | 28,824 | 7,093 |
| Contig hits against TMHMM | 7,685 | 19.40 | 7,685 | 1 |
| Contig hits against PIR | 597 | 1.51 | 599 | 336 |
| Contig hits against PRINTS | 2,042 | 5.16 | 2,313 | 378 |
| Contig hits against PROFILE | 6,208 | 15.67 | 7,484 | 491 |
| Contig hits against SMART | 4,692 | 11.85 | 6,109 | 485 |
| Contig hits against SIGNALP | 7,775 | 19.63 | 7,775 | 1 |
| Contig hits against PROFITE | 2,918 | 7.37 | 3,549 | 578 |
| Contig hits against TIGRFAMs | 1,577 | 3.98 | 1,684 | 580 |
