# Supplementary figures and images for: Comparative transcriptome analysis of the interaction between Actinidia chinensis var. chinensis and Pseudomonas syringae pv. actinidiae in absence and presence of acibenzolar-S-methyl
Source: BMC Genomics. 2018 Aug 6;19:585. doi: 10.1186/s12864-018-4967-4 (PMC6090863; doi:10.1186/s12864-018-4967-4)

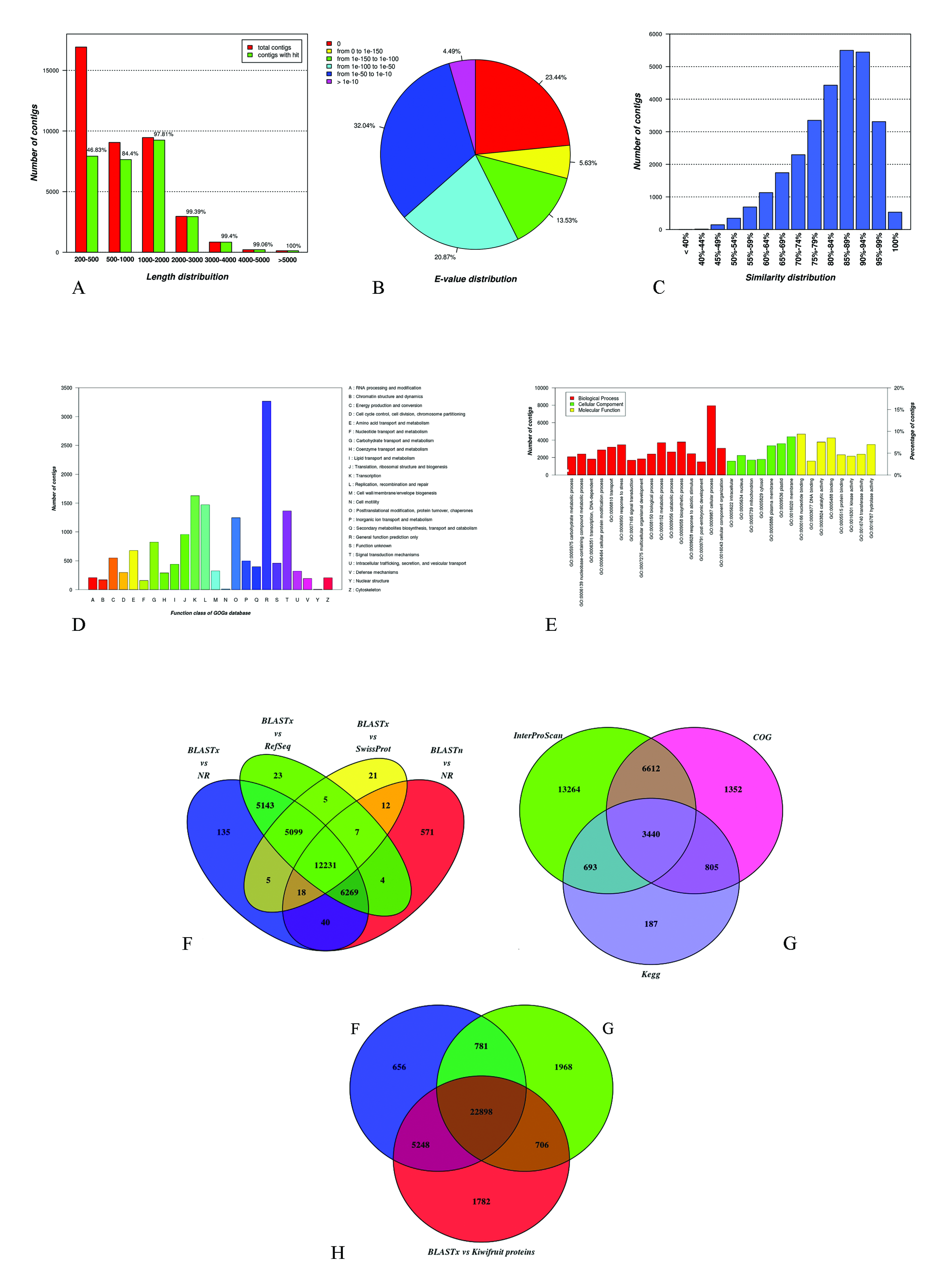

Supplement: Supplementary file 3 — Figure S1. Main characteristics of the annotation of the Actinidia chinensis var. chinensis reference transcriptome. Panel A: contig distribution by length. Panel B: contigs distribution by E-value. Panel C: contig distribution by similarity. Panel D The contigs of the A. chinensis var. chinensis reference transcriptome have been classified in Clusters of Orthologous Groups (COGs) functional annotations and organized into 24 function categories. Panel E Most relevant functional categories of the Groups of Orthologs obtained with the A. chinensis var. chinensis transcriptome. Venn diagrams illustrating the distribution of similarity search results made with the A. chinensis var. chinensis transcriptome. In panel F, the BLAST results against NR protein, NR nucleotide, RefSeq and SwissProt databases are reported. The total number of annotations obtained from these databases was 29,583 (74.73% of total contigs). In panel G are shown the annotation results obtained from COGs, InterProScan and KEGG. The number of contigs showing a hit against these databases was 26,353 (66.57% of total contigs). Panel H reports the intersection among the annotations presented in A and B with the results obtained from the BLASTx search against the Kiwifruit Genome protein database. Summarizing the results obtained from all the queried databases, a total of 34,039 contigs (85.99% of total contigs) were annotated. (TIF 2577 kb) [file 12864_2018_4967_MOESM3_ESM.tif]

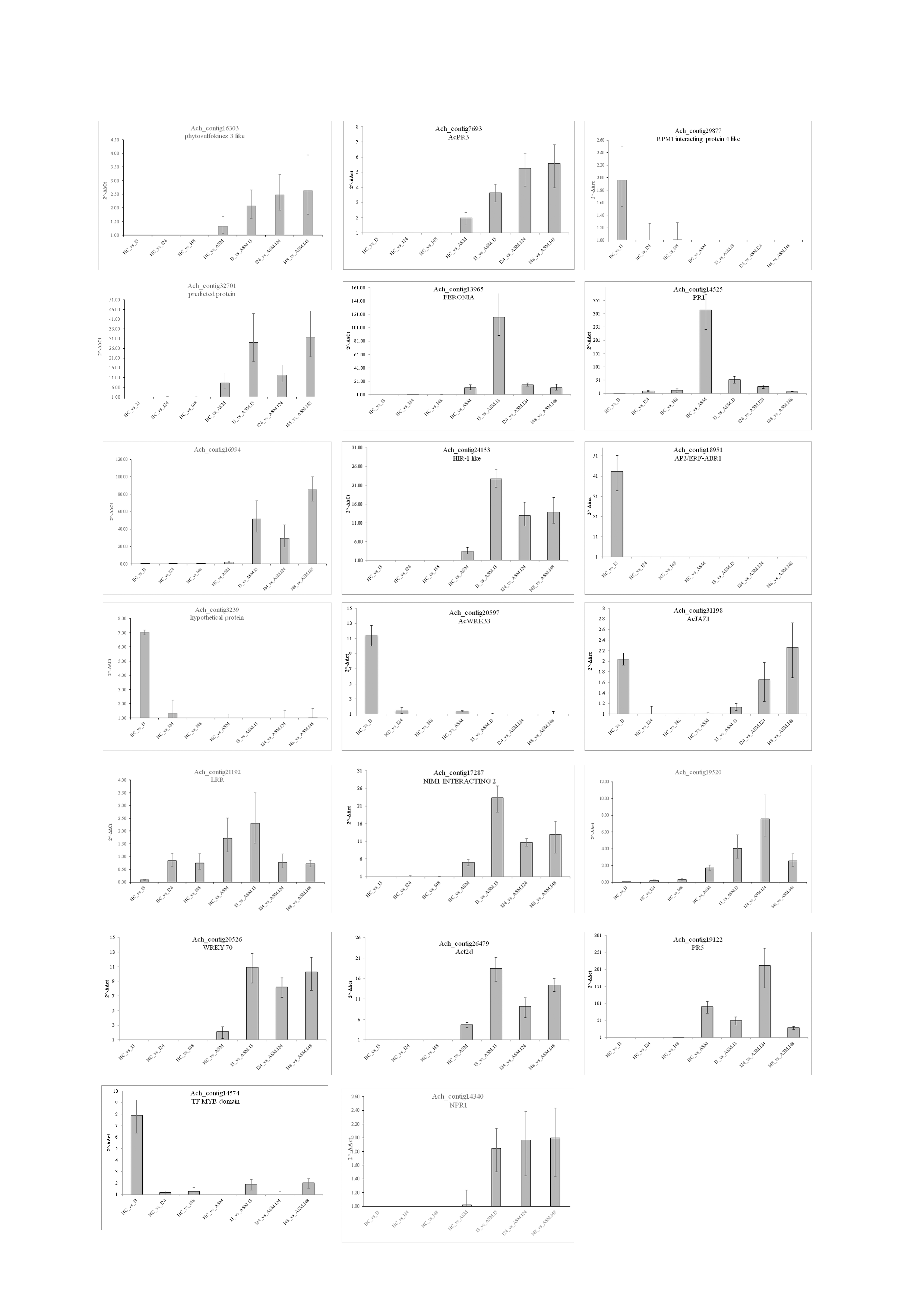

Supplement: Supplementary file 5 — Figure S2. qRT-PCR analysis was employed to validate the expression of twenty differentially expressed genes (DEGs). Ach_contigs and primers used are reported in Additional file: Table ST1, the PCR conditions are described in Material and Methods. Gene expression expressed as fold change and time course is indicated in the X axis. Standard errors are indicated. Results of quantitative PCRs were in agreement with RNA-seq experiment. (TIF 1088 kb) [file 12864_2018_4967_MOESM5_ESM.tif]

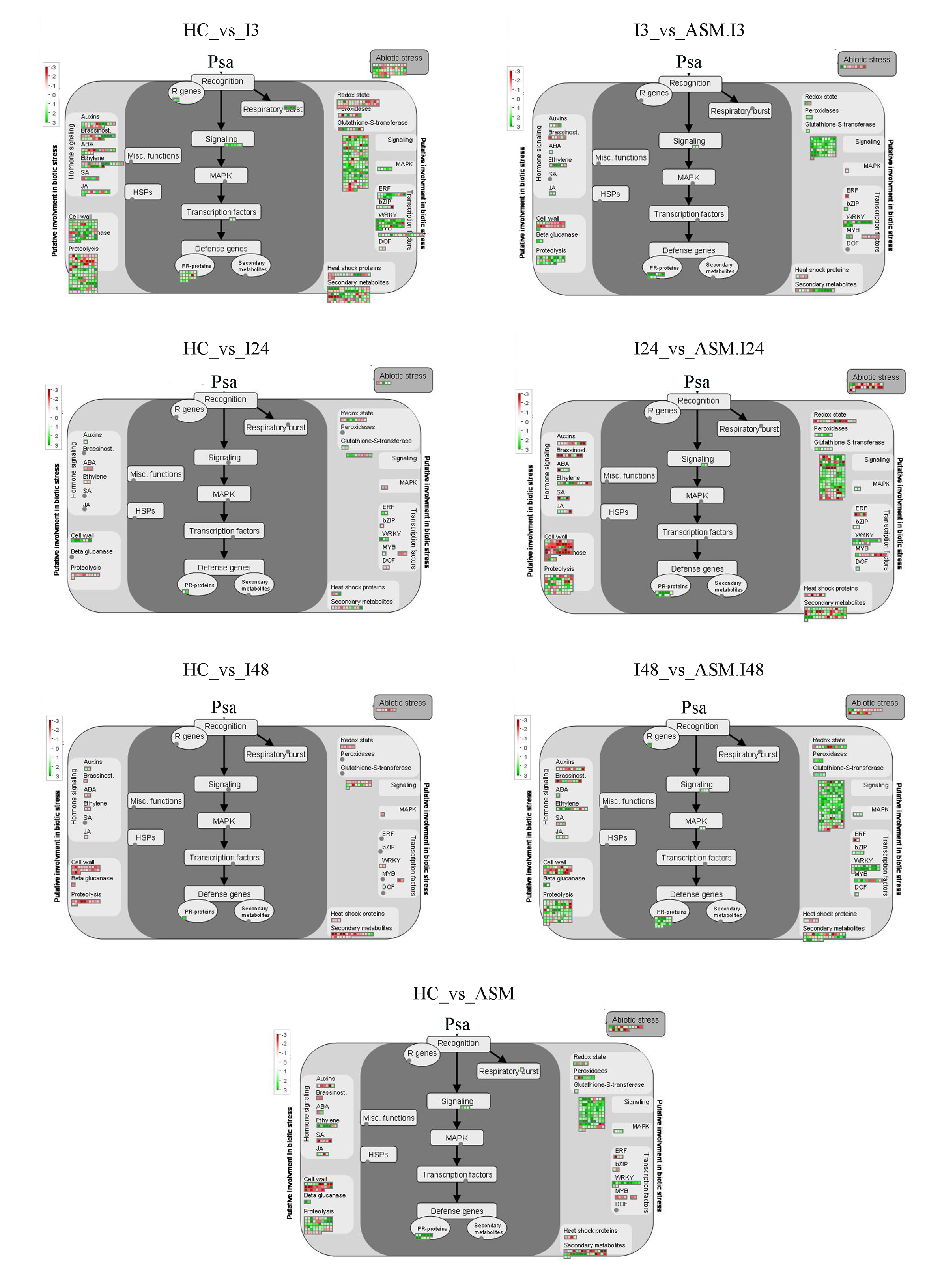

Supplement: Supplementary file 6 — Figure S3. The MapMan figure of the “Biotic stress” was obtained by running the Mercator tool (http://mapman.gabipd.org/web/guest/mercator) with default parameters to assign MapMan bins to Actinidia chinensis var. chinensis transcripts. (TIF 3354 kb) [file 12864_2018_4967_MOESM6_ESM.tif]

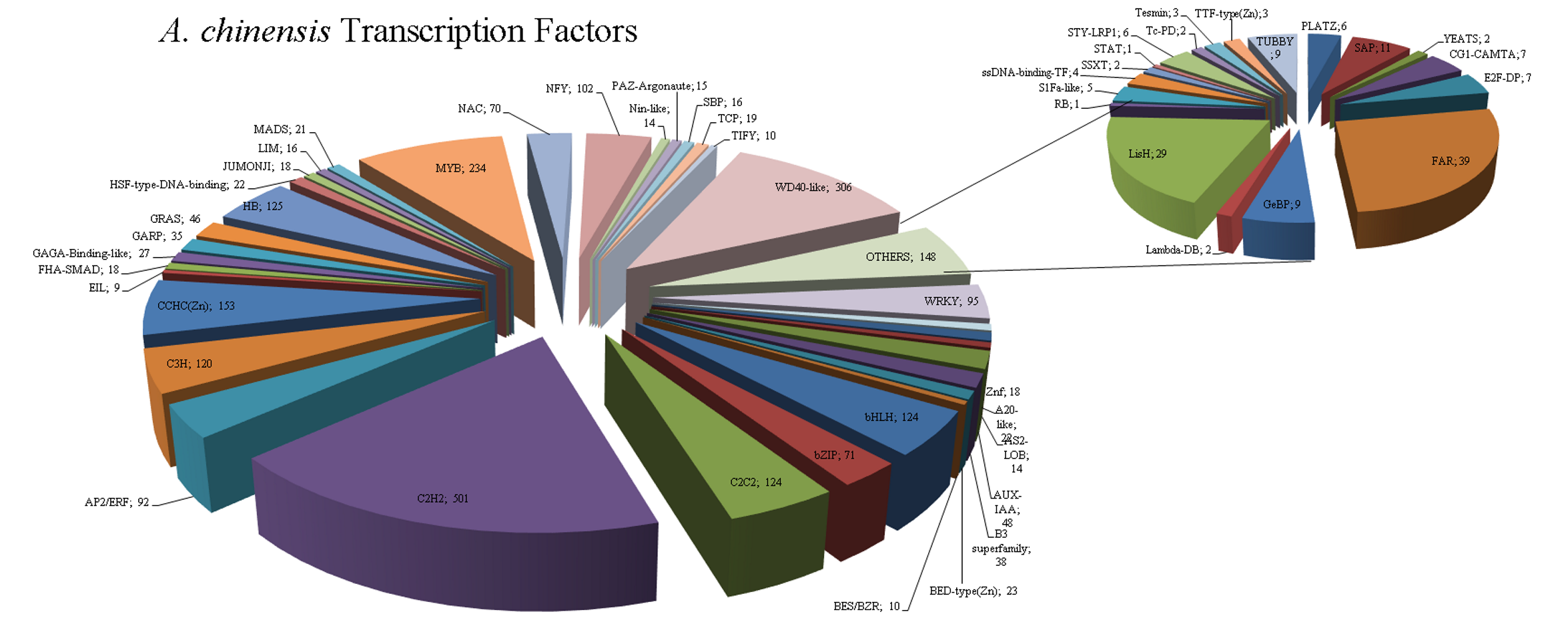

Supplement: Supplementary file 15 — Figure S4. Distribution of transcription factors (TFs) in Actinidia chinensis var. chinensis transcriptome based on BLASTx against plantTFDBcat (http://plantgrn.noble.org/PlantTFcat). Chromatin remodelling factors and families of TF with less than 5 transcripts are not shown. C2C2 family harbours: CO-like Dof, GATA, LSD and YABBY TFs. AP2/ERF includes the AP2, ERF and RAV classes of TFs. ARF and B3 classes belong to B3 superfamily of TFs. HD-ZIP, TALE, WOX, HB-PHD, and HB-other were grouped in the HB class. GARB family of TFs consists of ARR and G2-like classes. Others category consists of FHA-SMAD, GAGA-Binding-like, GeBP, HSF-type-DNA-binding, Nin-like, PAZ-Argonaute, PLATZ, Znf-B, Znf-LSD, SAP, SBP, TCP, TUBBY, FAR, CG1-CAMTA, E2F-DP, STY-LRP1, CW-Zn. Numbers of transcripts for each family were summarized in the figure and are detailed in Additional file: Table S12. (TIF 750 kb) [file 12864_2018_4967_MOESM15_ESM.tif]

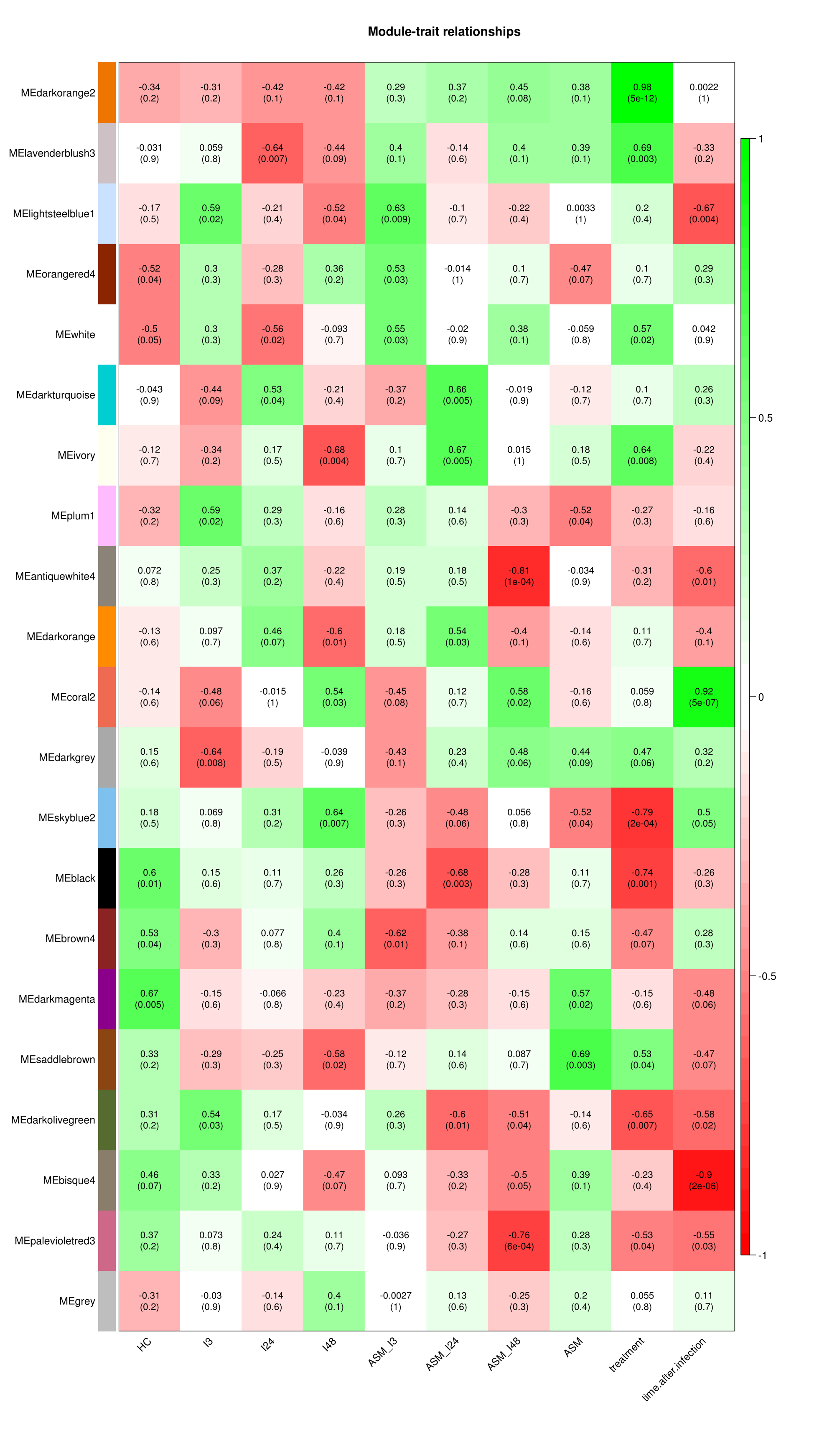

Supplement: Supplementary file 22 — Figure S5. Heatmap of the correlation of WGCNA modules with traits (ASM treatment and Psa inoculation). 21 modules were detected and named with colour names. The grey category is not a real module: it collect all the leftover genes not enough correlated with one of the other significant coloured modules. In each square the upper value is kME (module eigengene-based connectivity) while the lower value is the P-value of the correlation. (TIF 1752 kb) [file 12864_2018_4967_MOESM22_ESM.tif]
